# SGLT2 Inhibitors and the Risk of Arrhythmias in Heart Failure: A Network Meta-Analysis

**DOI:** 10.3390/jcm14155306

**Published:** 2025-07-27

**Authors:** Suchith Boodgere Suresh, Aishwarya Prasad, Muhammad Furqan Ubaid, Saad Farooq, Adrija Hajra, Vikash Jaiswal, Aaqib Malik, Gregg C. Fonarow, Dhrubajyoti Bandyopadhyay

**Affiliations:** 1 Internal Medicine, Montefiore St Luke’s Cornwall, Newburgh, NY 12550, USA; aishprasad0@gmail.com (A.P.); muhammadfubaid@gmail.com (M.F.U.); farooqsaadmd@gmail.com (S.F.); 2 Internal Medicine, Brigham and Women’s Hospital, Harvard Medical School, Boston, MA 02115, USA; adrija847@gmail.com; 3 Cardiology, Larkin Community Hospital, South Miami, FL 33143, USA; vikash29jaxy@gmail.com; 4 Cardiology, Catholic Medical Center, Manchester, NH 03102, USA; aaqib.malik@hcahealthcare.com; 5 Cardiology, UCLA, CA 90095, USA; gfonarow@mednet.ucla.edu; 6 Cardiology, Massachusetts General Hospital, Harvard Medical School, Boston, MA 02115, USA; drdhrubajyoti87@gmail.com

**Keywords:** sudden cardiac death, atrial fibrillation, sodium-glucose cotransporter-2 inhibitor, arrhythmia

## Abstract

**Background/Objectives:** Sodium-glucose cotransporter-2 inhibitors (SGLT2i) have revolutionized heart failure (HF) therapies and are an essential component of guideline-directed medical therapy (GDMT); however, their significance in arrhythmia prevention is still uncertain. This meta-analysis evaluates the benefits of SGLT2i on arrhythmias in HF. **Methods:** A comprehensive examination was performed with PubMed, ScienceDirect, PLOS One, Cochrane, Google Scholar, and ClinicalTrials.gov from January 2014 to March 2025, complying with PRISMA guidelines. Randomized controlled trials (RCTs) comparing SGLT2i with placebo were incorporated. Primary results included ventricular arrhythmias (VA), sudden cardiac death (SCD), atrial arrhythmias, and conduction disorders. Subgroup analyses investigated the effects on arrhythmias in HF with reduced ejection fraction (HFrEF) and preserved ejection fraction (HFpEF). **Results:** A total of 11 RCTs involving 23,701 patients, 11,848 on SGLT2i (mean age: 68.26 ± 10 yrs, 53.5% males) and 11,853 on placebo (mean age: 67.91 ± 10 yrs, 53% males), were analyzed with a mean follow-up of 2.71 yrs. No significant differences were reported between SGLT2i and placebo for VA [relative risk (RR): 1.02, 95% confidence interval (CI): 0.83–1.25], I^2^ =0%), atrial arrhythmias (RR: 0.92 [CI: 0.67–1.27], I^2^ = 65.3%), or conduction disorders (RR:1.22 [CI: 0.86–1.73], I^2^ = 10.4%). Notably, significant reductions in risk of SCD (RR: 0.68 [CI: 0.49–0.93], I^2^ = 0%) and in the risk of atrial arrhythmias in HFrEF (RR: 0.66 [CI: 0.49–0.89], I^2^ = 10.3%) were witnessed, although no such reduction was seen in HFpEF (RR: 1.14 [CI: 0.94–1.40], I^2^ = 33.8%). **Conclusions:** SGLT2i do not reduce overall arrhythmia or conduction disorder risk in HF but significantly reduce the risk of SCD and atrial arrhythmias in HFrEF patients. These results highlight potential arrhythmia prevention benefits in HFrEF, warranting further targeted studies.

## 1. Introduction

Heart failure (HF) is a worldwide public health burden, with growing epidemiological and economic implications, impacting almost 64 million people globally and significantly contributing to cardiovascular morbidity and mortality [1]. Nearly 6.7 million adults in the US alone suffer from HF, making it the leading cause of hospitalization under Medicare, accounting for 9% of cardiac deaths [2]. The condition is often complicated by arrhythmias, which significantly impact patient outcomes and quality of life.

The Framingham Heart Study determined that atrial fibrillation (AF), a prevalent comorbidity in HF, occurred at an incidence of 54 cases per 1000 person-years [3]. Up to 35% of patients with HF with preserved ejection fraction (HFpEF) may experience non-sustained ventricular tachycardia (VT), suggesting the serious risk of ventricular arrhythmias (VA) in this population. Around one-quarter to one-third of fatalities in HF with reduced ejection fraction (HFrEF) are caused by sudden cardiac death (SCD), emphasizing the essential role that arrhythmias play in HF mortality [4]. HF occurs when the heart fails to pump enough blood to satisfy the requirements of the body. This pathophysiology can occur in two ways: due to heart muscle weakness and loss of ability to pump blood forward (in HFrEF) or stiffness in the heart, which reduces the volume of blood filling the ventricle (in HFpEF). Either of these leads to backflow, congestion, and fluid retention [1].

While angiotensin-converting enzyme Inhibitors (ACEi), angiotensin receptor-neprilysin inhibitors (ARNIs), angiotensin II receptor blockers (ARBs), beta-blockers, and mineralocorticoid receptor antagonists (MRAs) have long been the cornerstone of HF management, sodium-glucose co-transporter-2 inhibitors (SGLT2i) are now increasingly recognized as a key component of guideline-directed medical therapy. All of these act by inhibiting the renin–angiotensin–aldosterone system [4]. SGLT2i have been shown to improve HF mortality and morbidity, although their effect on arrhythmic events continues to be investigated [5].

**Figure 1:**
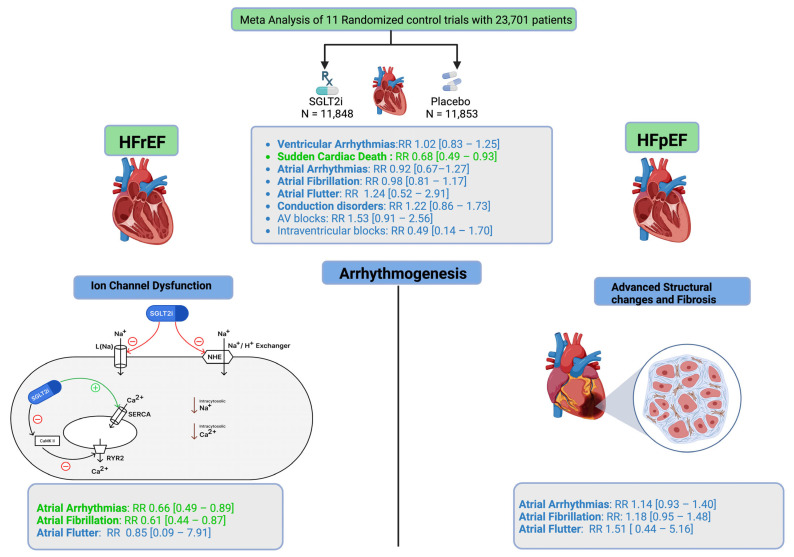
Ref. [6]: Meta-analysis of 11 randomized controlled trials demonstrates that SGLT2 inhibitors and the risk of arrhythmias, with subgroup analyses across heart failure phenotypes. The illustration highlights underlying mechanisms on ion channel modulation and structural remodeling. Created in BioRender. B suresh, S. (2025) https://BioRender.com/xvkacqi (26 July 2025).

The Central Illustration can be seen in Figure 1 [6].

Preclinical studies have suggested potential anti-arrhythmic effects of SGLT2i, possibly mediated through left ventricular remodeling, modulation of late sodium current channels, and regulation of the sodium-hydrogen exchanger [6]. However, the extent of these effects in HF patients with various arrhythmias and their clinical significance remain unclear.

This meta-analysis examines data from eleven double-blind, placebo-controlled randomized trials. It comprehensively evaluates the impact of SGLT2i on VA, atrial arrhythmia, SCD, and heart block outcomes. Our goal is to provide evidence to inform clinical decision making and potentially reshape the development of arrhythmia management strategies for individuals with HF.

## 2. Methods

This network meta-analysis was conducted and reported in accordance with the PRISMA-NMA (Preferred Reporting Items for Systematic Reviews and Meta-Analyses Extension for Network Meta-Analysis) guidelines.

### 2.1. Data Collection Process

**Figure 2:**
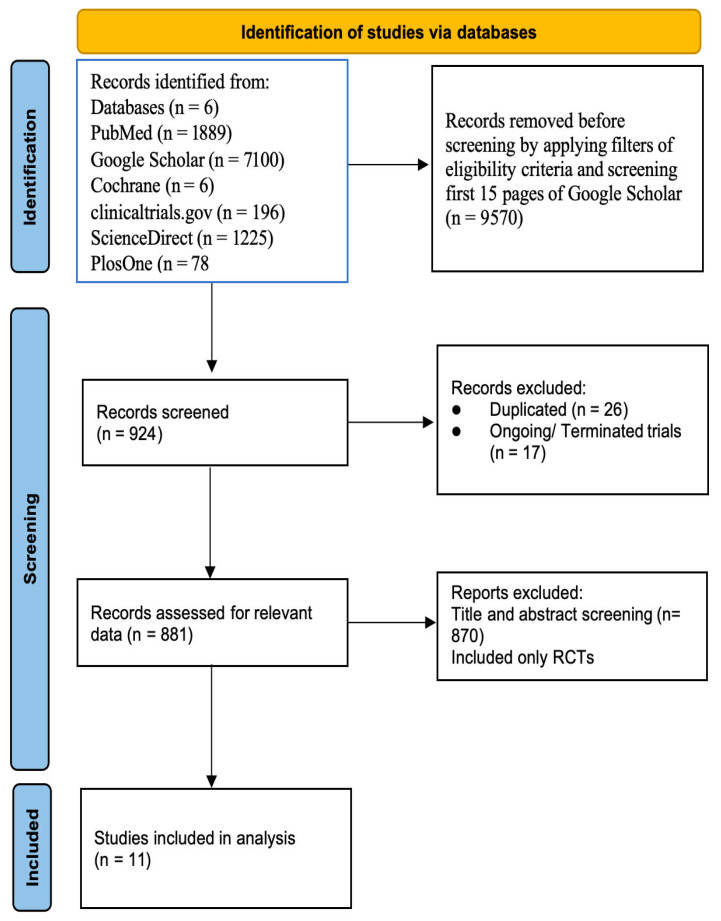
PRISMA flowchart.

Between January 2014 and March 2025, we conducted a comprehensive search of PubMed, Google Scholar, PLOS One, Cochrane, ClinicalTrials.gov., and ScienceDirect. The search strategy is described in Table S1 (Supplementary Data). Our systematic search identified 10,494 records across multiple databases. Title and abstract screening was conducted on 898 unique records following application of filters and elimination of duplicates. A total of 870 were excluded, considering they were irrelevant. From the remaining 28 full-text articles assessed, 17 were excluded (ongoing/terminated trials). Eleven RCTs met the inclusion criteria (Figure 2). The Revised Cochrane Risk of Bias Tool for Randomized Trials (RoB 2.0) tool was employed for evaluating the quality of the articles (Table 1).

### 2.2. Study Selection

The articles were imported into Rayyan AI from each of the above databases. Titles and abstracts of every publication that was retrieved were initially checked by two different reviewers to ensure that they fulfilled eligibility requirements (Table 2). The two-stage study selection procedure commenced at this stage. Discussions and consulting a third reviewer were employed to resolve any disagreements. Subsequently, during full-text review, the second group of reviewers separately assessed every text in potentially relevant research. Any disagreements were resolved by the agreement of all three reviewers.

### 2.3. Data Extraction

Trial characteristics, baseline demographics, and outcomes were extracted using a pre-designed Excel spreadsheet. The primary outcomes assessed included VA, SCD, atrial arrhythmia, and conduction disorders. Secondary outcomes included VT, ventricular fibrillation (VF), AF, atrial flutter (AFL), sinoatrial node (SAN) dysfunction, atrioventricular conduction block (AVB), and intraventricular conduction block (IVB). Subgroup analysis was performed based on heart failure types and drug types.

### 2.4. Data Analysis

#### 2.4.1. Pairwise Meta-Analysis

We performed a pairwise meta-analysis using the inverse variance method implemented in the ‘meta’ package in R (Version 4.4.2 (31 October 2024)). For binary outcomes, we used the ‘metabin’ function with relative risk (RR) as the measure of effect. The inverse variance method was employed, which weights studies according to the inverse of the variance of the effect estimate, thus giving more weight to larger studies with smaller standard errors. Between-study heterogeneity was accounted for using the restricted maximum likelihood (REML) estimator for the between-study variance (τ^2^). For each comparison, we presented both fixed-effects and random-effects models, with the latter serving as our primary analysis given the clinical diversity across included studies. *p* values < 0.05 were considered statistically significant.

Heterogeneity was quantified using the I^2^ statistic, with values of 0–25%, 26–50%, and >50% considered as low, moderate, and high heterogeneity, respectively. We conducted subgroup analyses based on heart failure type (HFrEF, HFpEF, or both) and specific SGLT2i (Sotagliflozin, Empagliflozin, Canagliflozin, or Dapagliflozin).

#### 2.4.2. Network Meta-Analysis

To compare the relative effects of SGLT2i, we conducted a frequentist network meta-analysis using the ‘netmeta’ package in R (Version 4.4.2 (31 October 2024)). We transformed the arm-based data into a contrast-based format using the ‘pairwise’ function, with placebo serving as the common comparator across all trials. Employing both common-effect and random-effects models, the method combines direct evidence (from head-to-head comparisons) and indirect evidence (derived from studies with a common comparator) to estimate all pairwise comparisons between treatments. Network geometry shows treatment connections using nodes (circles) for treatments and lines for direct comparisons. Line thickness indicates study count, visualizing evidence strength.

### 2.5. Inconsistency Assessment

To evaluate the agreement between direct and indirect evidence, we employed the ‘netsplit’ function with the “show = all” parameter to assess consistency across the network. This approach splits the network estimates into direct and indirect components and tests for local inconsistency. The results were visualized using forest plots showing direct, indirect, and network estimates for each pairwise comparison, along with tests of inconsistency (Supplementary Data).

### 2.6. Treatment Ranking

Treatments were ranked with P-scores, a frequentist metric in network meta-analysis that summarizes both direct and indirect evidence. A P-score ranges from 0 (least effective) to 1 (most effective) and reflects the average certainty that a given treatment outperforms each of its competitors across the entire network. Rankings were displayed visually using the netrank function. Heatmaps use color gradients to rank treatment effectiveness based on P-scores values, with darker colors indicating higher incidence of arrhythmia.

### 2.7. League Table Synthesis

Pairwise treatment comparisons were structured using ‘netleague’ to generate league tables displaying network estimates (relative risk with 95% CIs) for all SGLT2 inhibitor vs. placebo comparisons. The lower triangle presented network meta-analysis results, while the upper triangle displayed direct evidence estimates. Tables were sorted by descending P-scores and exported in matrix format, incorporating both common-effect and random-effects models (Supplementary Data).

## 3. Results

Eleven RCTs encompassing 23,701 patients, with 11,848 on SGLT2i (mean age: 68.26 ± 10 yrs, 53.5% males) and 11,853 on placebo (mean age: 67.91 ± 10 yrs, 53% males), were analyzed with a mean follow-up of 2.71 yrs (Table 3).

**Figure 3:**
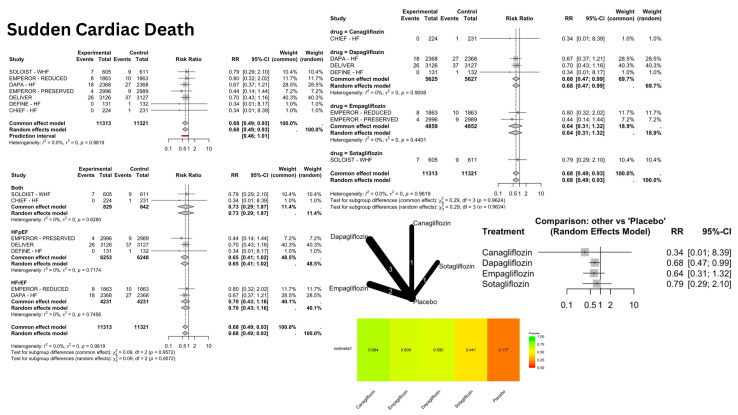
Risk of sudden cardiac death (SCD) with SGLT2 inhibitors: Forest plots display risk ratios for SCD overall, stratified by heart failure phenotypes and specific SGLT2 inhibitors (Dapagliflozin, Empagliflozin, Canagliflozin, Sotagliflozin) versus placebo. The network diagram illustrates direct and indirect comparisons among these treatments. The heatmap shows the relative ranking of each treatment based on netmeta analysis.

**Figure 4:**
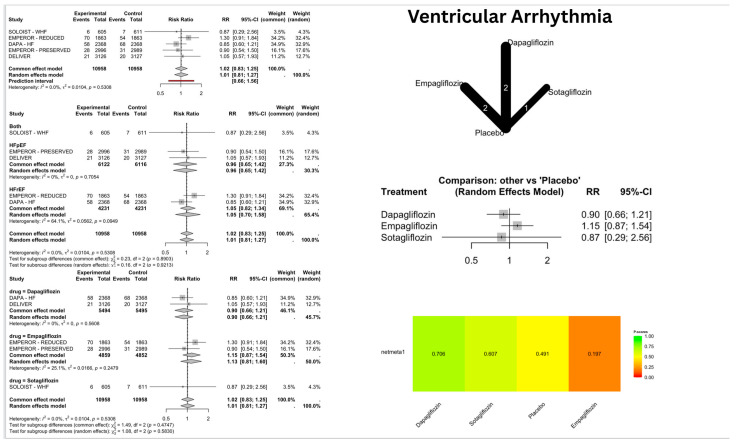
Risk of ventricular arrhythmia (VA) with SGLT2 inhibitors: Forest plots display risk ratios for VA overall, stratified by heart failure phenotypes and specific SGLT2 inhibitors (Dapagliflozin, Empagliflozin, Sotagliflozin) versus placebo. The network diagram illustrates direct and indirect comparisons among these treatments. The heatmap shows the relative ranking of each treatment based on netmeta analysis.

**Figure 5:**
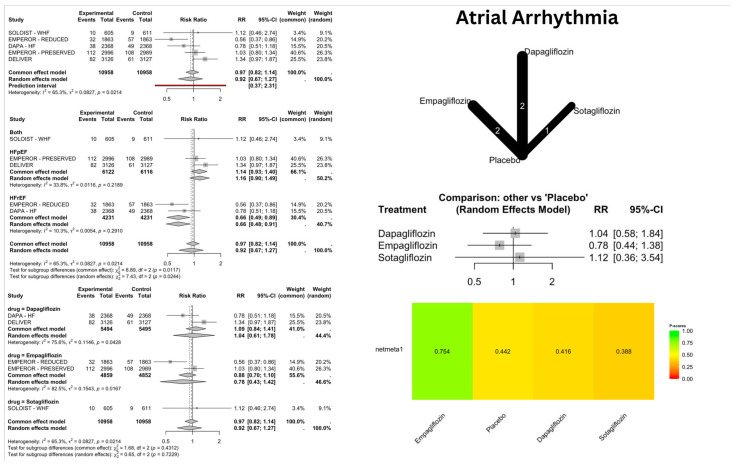
Risk of atrial arrhythmia (AA) with SGLT2 inhibitors: Forest plots display risk ratios for AA overall, stratified by heart failure phenotypes and specific SGLT2 inhibitors (Dapagliflozin, Empagliflozin, Sotagliflozin) versus placebo. The network diagram illustrates direct and indirect comparisons among these treatments. The heatmap shows the relative ranking of each treatment based on netmeta analysis.

**Figure 6:**
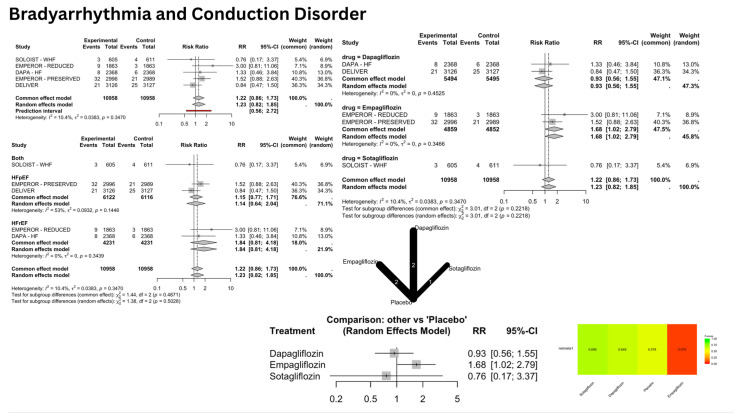
Risk of bradyarrhythmia and conduction disorder with SGLT2 inhibitors: Forest plots display risk ratios for bradyarrhythmia and conduction disorder overall, stratified by heart failure phenotypes and specific SGLT2 inhibitors (Dapagliflozin, Empagliflozin, Sotagliflozin) versus placebo. The network diagram illustrates direct and indirect comparisons among these treatments. The heatmap shows the relative ranking of each treatment based on netmeta analysis.

SGLT2i showed a significant reduction in the risk of SCD [Figure 3], with a pooled relative risk (RR) of 0.68 [confidence interval (CI): 0.49–0.93]; *p* = 0.016, I^2^ = 0%. However, the arrhythmic outcomes—VA [Figure 4], VF [Figure S1], VT [Figure S2], AA [Figure 5], AF [Figure S3], AFL [Figure S4], bradyarrhythmias and conduction disorders [Figure 6], SAN dysfunction [Figure S5], AVB [Figure S6], and IVB [Figure S7]—showed no significant difference compared to placebo.

### 3.1. Heart Failure Phenotype-Specific Effects

#### 3.1.1. Heart Failure with Preserved Ejection Fraction (HFpEF)

In HFpEF patients (12,238–12,877 participants), SGLT2i showed no significant reduction in VA (RR: 0.96, *p* = 0.841), AF (RR: 1.18, *p* = 0.141), or SCD (RR: 0.65, *p* = 0.061), with no impact on bradyarrhythmias/conduction disorders.

#### 3.1.2. Heart Failure with Reduced Ejection Fraction (HFrEF)

In HFrEF (8462 to 9036 participants), SGLT2i demonstrated neutral effects on most arrhythmic outcomes, including VA, VF, VT, AVB, IVB, AFL, SCD, bradyarrhythmias/conduction disorders, and SAN dysfunction, with no significant differences vs. placebo. However, two significant antiarrhythmic benefits emerged: a reduction in AA (RR: 0.66, 95% CI: 0.49–0.89; *p* = 0.007; I^2^ = 10.4%) and AF (RR: 0.61, 95% CI: 0.44–0.87; *p* = 0.005; I^2^ = 0%).

### 3.2. Drug-Specific Effects (Table 4)

#### 3.2.1. Dapagliflozin

Dapagliflozin demonstrated beneficial effects specifically for SCD, showing a significant 32% risk reduction with an RR of 0.68 [0.47–0.99], I^2^ = 0%; *p* = 0.0471. However, the drug also presented adverse effects, particularly for AFL, which showed significantly increased risk with an RR of 3.03 [1.18–7.75], I^2^ = 0%; *p* = 0.021. The drug showed neutral effects for VA, AF, and conduction disorders.

#### 3.2.2. Empagliflozin

Empagliflozin was associated with several adverse effects, particularly in conduction-related outcomes. Bradyarrhythmias and conduction disorders showed significantly increased risk with an RR of 1.68 [1.02–2.79], I^2^ = 0%; *p* = 0.041. AVB demonstrated a significantly increased risk with a relative risk of 2.39 [1.14–5.03], I^2^ = 0%; *p* = 0.021. The drug showed neutral effects for VA, AF, and SCD.

#### 3.2.3. Sotagliflozin

Sotagliflozin consistently demonstrated non-significant effects across all arrhythmic outcomes analyzed in the meta-analysis. The analyses for this drug were characterized by wide confidence intervals in most outcomes, which limited the interpretability of the results.

#### 3.2.4. Canagliflozin

Canagliflozin had limited data available for analysis, with most outcomes characterized by wide confidence intervals that reduced the reliability of the findings. No significant effects were demonstrated in the limited analyses that could be performed for this drug.

### 3.3. Network Meta-Analysis (NMA) Results

The NMA provided comparative effectiveness rankings while confirming the individual drug effects observed in the direct comparisons. For SCD, the P-score rankings showed Canagliflozin at 0.68 (though this was unreliable due to wide confidence intervals), Empagliflozin at 0.61, and Dapagliflozin at 0.59, with Dapagliflozin being the only statistically significant treatment. In AF outcomes, Empagliflozin achieved the highest ranking with a P-score of 0.67, though this was non-significant, while placebo scored 0.44, and Dapagliflozin, along with other treatments, received lower rankings.

For AFL, Empagliflozin demonstrated the best safety profile with a P-score of 0.86, whereas Dapagliflozin ranked lowest at 0.19 due to a substantial increase in risk. In bradyarrhythmia outcomes, Sotagliflozin achieved the best ranking with a P-score of 0.70, although this was unreliable due to the wide confidence intervals, while Empagliflozin had the poorest ranking at 0.08, due to its significant risk increase.

### 3.4. NMA Consistency and Heterogeneity

The network meta-analyses demonstrated robust methodological quality with generally low heterogeneity across most outcomes, ranging from 0% to 5.8% (I^2^). The analyses showed consistent results between direct and indirect comparisons, supporting the validity of the network approach. The findings were further supported by minimal inconsistency between study designs, enhancing confidence in the results [Supplementary Data 3].

The clinical interpretation emphasizes that while P-score rankings provide useful relative positioning of treatments, only statistically significant differences should guide clinical decision making. This approach highlights Dapagliflozin’s benefit for sudden cardiac death prevention and its associated risk for AFL, alongside Empagliflozin’s risks for various conduction disorders.

## 4. Discussion

This NMA explored SGLT2i effects on arrhythmia-related outcomes in HF patients, revealing important phenotype-specific and agent-specific differences. While SGLT2i did not reduce VA risk, they significantly reduced SCD (RR: 0.68 [0.49–0.93], *p* = 0.02), with consistent effects across DAPA-HF (18/2368 vs. 27/2368), DELIVER (26/3126 vs. 37/3127), EMPEROR-Reduced (8/1863 vs. 10/1863), and EMPEROR-Preserved (4/2996 vs. 9/2989). In HFrEF patients specifically, SGLT2i reduced atrial arrhythmias (RR: 0.66 [0.49–0.89], *p* = 0.007) and AF (RR: 0.61 [0.44–0.87], *p* = 0.0053), consistent with pooled data from DAPA-HF (38/2368 vs. 49/2368) and EMPEROR-Reduced (32/1863 vs. 57/1863). Notable agent-specific differences emerged, with Dapagliflozin increasing the risk of AFL (RR: 3.03 [1.18–7.75], *p* = 0.02) in DAPA-HF (8/2368 vs. 3/2368) and DELIVER (10/3126 vs. 2/3127), whereas Empagliflozin showed a protective trend in EMPEROR-Reduced (3/1863 vs. 11/1863). These findings suggest that SGLT2i may offer selective arrhythmia protection with important differences among individual agents that should inform clinical decision making.

**Figure 7:**
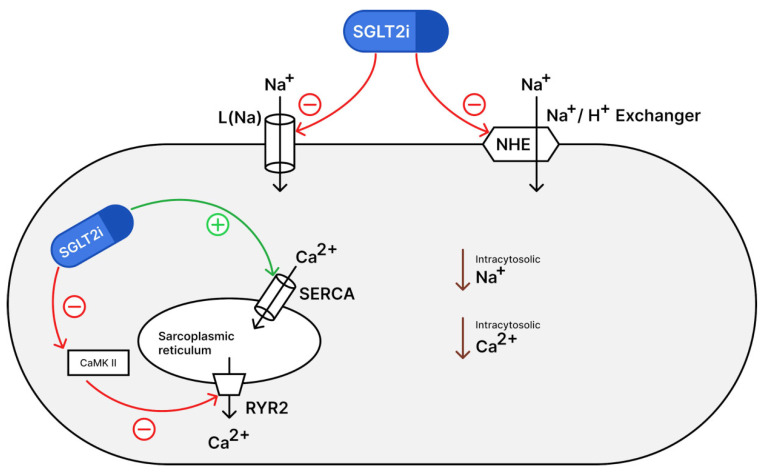
Electrophysiological mechanisms of SGLT2i [6].

Preclinical studies have shown how SGLT2i modulate electrophysiology in cardiac myocytes (Figure 7). They inhibit late sodium current channels (INa, L) and sodium-hydrogen exchanger (NHE). This reduces intracytosolic sodium, which also prevents the activation of sodium-calcium exchange, thereby reducing intracytosolic calcium. Concurrently, SGLT2i enhance sarcoplasmic reticulum calcium ATPase (SERCA2a), facilitating calcium reuptake; they also suppress “calcium/calmodulin-dependent protein kinase II (CaMKII)” activity, which reduces ryanodine receptor (RyR2) hyperphosphorylation and prevents the release of calcium into the cytoplasm [6]. Because I Na, L is increased in both atrial fibrillation (AF) and Long QT Syndrome 3, SGLT2i’s late I Na blockade may potentially provide therapeutic benefit in AF [18].

Together, these mechanisms attenuate both early afterdepolarizations (EADs) and delayed afterdepolarizations (DADs), the predominant cellular triggers for atrial and VA in heart failure. In HFrEF, where arrhythmogenesis is primarily driven by neurohormonal activation and ion channel dysfunction, these electrophysiological effects have a greater impact compared to HFpEF, where advanced structural changes and fibrosis are the foci of arrhythmogenesis. This mechanistic distinction aligns with our meta-analysis findings showing significant atrial arrhythmia reduction in HFrEF (34% reduction) but not in HFpEF patients.

SGLT2i reduce cardiac workload through natriuresis and improve left ventricular function. They also exert anti-fibrotic actions, promoting M2 macrophage polarization, suppressing TGF-β-mediated collagen synthesis, and inhibiting myofibroblast activation. By attenuating myocardial fibrosis, which underlies reentry circuits in both atrial and ventricular arrhythmias, SGLT2i prevent arrhythmogenesis in animal models of heart failure [18].

SGLT2i attenuate sympathetic nervous system activity through both direct and indirect pathways: they suppress catecholamine synthesis by downregulating tyrosine hydroxylase expression in cardiac and renal tissues, leading to reduced norepinephrine and epinephrine levels; elevate circulating β-hydroxybutyrate, which blocks FFAR3 on sympathetic nerve terminals and thereby diminishes norepinephrine release; restore normal renal tubuloglomerular feedback via enhanced natriuresis and osmotic diuresis, reducing renal afferent nerve firing and central sympathetic outflow [18]. These mechanisms help reduce the propensity for arrhythmias.

Our NMA expands upon previous work by Liao et al. [19] and Wang et al. [20], incorporating three additional trials (DELIVER, PRESERVED-HF, and CHIEF-HF) and conducting more comprehensive analyses, including network meta-analysis with P-scores and rankograms. Unlike previous analyses, we focused exclusively on heart failure patients and stratified results by heart failure phenotype (HFrEF vs. HFpEF) and specific SGLT2i.

### 4.1. Atrial Arrhythmias

Our analysis demonstrated a 39% reduction in AF risk in HFrEF patients, which exceeds the 34% reduction reported by Liao et al. and is consistent with the findings by Fedele et al. (RR: 0.62) [21]. However, findings for HFpEF patients remained non-significant, consistent with previous studies by Liao et al. and Fedele et al. Contrary to Liao et al., who reported a significant risk reduction with Dapagliflozin (RR: 0.73 [0.63, 0.95]), our drug-specific network meta-analysis revealed no significant differences between SGLT2 inhibitors, with Empagliflozin achieving the highest P-score (0.67), while Dapagliflozin ranked lowest (0.43). This discrepancy likely stems from the inclusion of the DELIVER trial, where more AF events occurred in the treatment group than placebo (69 vs. 57), and the PRESERVED-HF trial (3 vs. 0).

AFL analysis showed consistency with prior research regarding phenotypic trends but revealed novel drug-specific risks. Dapagliflozin showed a 3.03-fold increased risk (*p* = 0.02), contrasting with Liao et al.’s non-significant inter-drug comparisons. The DELIVER trial reported a higher rate of AFL in treated than placebo (10 vs. 2).

### 4.2. Ventricular Arrhythmias and SCD

Our analysis expanded upon Liao et al.’s work by including ventricular flutter and torsade de pointes alongside VF, VT, and ventricular extrasystole. Overall, we found no significant risk differences for VA in HF patients, regardless of HF phenotype or specific SGLT2i used. These findings maintain consistency with previously published meta-analyses.

Our analysis demonstrated a 32% reduction in sudden cardiac death risk (RR: 0.68 [0.49–0.93], *p* = 0.02), which closely aligns with Liao et al.’s borderline significant finding of a 33% reduction (RR: 0.67 [0.44–1.01], *p* = 0.05). The enhanced statistical significance in our analysis likely results from greater statistical power due to the inclusion of the DELIVER and CHIEF trials.

Similar to Liao et al., our subgroup analyses showed no significant risk reduction in either HFpEF or HFrEF patients specifically. Our NMA identified a significant risk reduction with Dapagliflozin (RR: 0.68 [0.47–0.99], *p* = 0.047; P-score = 0.59), contrasting with Liao et al.’s non-significant finding for Dapagliflozin (RR: 0.73 [0.47–1.15]). In our analysis, Dapagliflozin ranked third after canagliflozin (P-score = 0.68) and Empagliflozin (P-score = 0.61).

### 4.3. Bradyarrhythmias and Conduction Disorders

Bradyarrhythmia analysis revealed nuanced drug-specific risks despite overall non-significant results, mirroring Wang et al.’s findings (RR: 0.92, *p* = 0.34). Network meta-analysis revealed a significantly increased risk of 68% with Empagliflozin (RR: 1.68, *p* = 0.04), which ranked worst with a P-score of 0.08 (following placebo, P-score = 0.58). Conduction disorder analyses showed divergent trends. SAN dysfunction risk remained non-significant (RR: 1.08, *p* = 0.81), consistent with Wang et al. (RR: 0.94, *p* = 0.71). Our findings regarding AVB were statistically non-significant, consistent with Wang et al. (RR: 1.14 [0.87–1.48], *p* = 0.35). However, our network meta-analysis identified a significantly higher risk with Empagliflozin (RR: 2.39 [1.14–5.03], *p* = 0.02), which ranked lowest with a P-score of 0.17. Our analysis of IVB showed non-significant results, consistent with Wang et al.’s findings (RR: 0.59 [0.28–1.24], *p* = 0.16).

### 4.4. Clinical Implications

This NMA offers important new evidence on the arrhythmia-related effects of SGLT2i in HF, highlighting clinically relevant differences by HF phenotype and individual drug. Notably, SGLT2i reduced AF risk by 39% in HFrEF patients (RR: 0.61), consistent with their favorable impact on cardiac electrophysiology in reduced ejection fraction. Dapagliflozin significantly reduced SCD (RR: 0.68) but increased AFL risk, while Empagliflozin was associated with a higher risk of AVB. These drug-specific findings go beyond previous class-level analyses and fill a gap in current guidelines, which do not address arrhythmia outcomes.

Clinically, these results support the use of SGLT2 inhibitors, particularly Empagliflozin and Dapagliflozin, in HF patients at risk for AF or SCD. However, given the observed trend toward a higher incidence of conduction disorders with Empagliflozin and AFL with Dapagliflozin in our analysis, although with low absolute event rates, careful monitoring is needed in patients with pre-existing conduction abnormalities (such as prolonged PR interval or high-degree AV block) when prescribing Empagliflozin, and in those with a predisposition to AFL when using Dapagliflozin. The neutral effect on VA across all phenotypes suggests SGLT2i are safe in this regard. By incorporating recent large trials and stratifying by heart failure type, this analysis strengthens the evidence base for individualized SGLT2i selection in heart failure patients with arrhythmia risk and highlights the need for further research in device populations and HFpEF, as well as trials focusing on arrhythmia risk, particularly in patients with HF.

## 5. Limitations

This analysis has several important limitations. First, arrhythmia events were secondary endpoints in the included trials, which prioritized heart failure hospitalizations and mortality as primary outcomes. The lack of mandated systematic electrocardiographic monitoring likely resulted in underdetection of asymptomatic arrhythmias, particularly paroxysmal AF and non-sustained VT. Second, while we stratified by heart failure phenotype, residual confounding persists due to heterogeneous baseline characteristics, comorbidities, and unmeasured variables such as antiarrhythmic drug use or implantable device therapies across trials. Third, publication bias and funnel plots could not be formally assessed for most outcomes due to the limited number of RCTs (<10). Fourth, while P-scores provided frequentist ranking probabilities, we did not calculate SUCRA metrics due to the rarity of several endpoints and the absence of Bayesian priors for indirect comparisons, limiting the interpretability of intervention hierarchies. Fifth, the analysis used study-level data rather than individual patient data, limiting our ability to conduct detailed subgroup analyses. This approach may introduce bias, where study-level findings do not accurately reflect individual patient responses. Sixth, the subgroup analyses for HFrEF and HFpEF, while clinically relevant, are underpowered and should be interpreted as exploratory rather than conclusive. Finally, despite pooling over 20,000 patients, statistical power remained insufficient for rare outcomes like AFL and specific conduction disorders, as evidenced by wide confidence intervals that preclude definitive conclusions. These limitations show the need for prospective trials with protocol-driven arrhythmia monitoring and standardized reporting of device-based rhythm data.

## 6. Conclusions

This NMA clarifies the antiarrhythmic profile of SGLT2i in HF, revealing distinct benefits and risks shaped by HF phenotype and specific agents. However, the drug-specific comparisons presented should be interpreted as hypothesis-generating rather than definitive clinical guidance due to the limited number of trials per individual agent.

The demonstrated reductions in AF risk among HFrEF patients align with the drugs’ electrophysiological mechanisms targeting sodium-calcium homeostasis, which mitigate arrhythmogenic triggers in neurohormonally active myocardium. In contrast, neutral effects in HFpEF show the limited impact of these pathways on structural arrhythmia substrates, emphasizing the need for phenotype-guided therapy. Agent-specific profiles further refine clinical decision making: while Dapagliflozin shows promise in reducing sudden cardiac death, its association with AFL risk warrants caution in predisposed patients, whereas Empagliflozin’s superior AF protection is tempered by heightened susceptibility to bradyarrhythmias and conduction disorders. The class-wide neutral VA profile reinforces cardiovascular safety across heart failure subtypes, particularly reassuring for patients with implantable devices.

These findings advocate for SGLT2i as first-line adjuvants for arrhythmia prevention in HFrEF, particularly for AF mitigation, highlighting the importance of tailoring agent choice according to individual arrhythmia profiles and conduction system characteristics. Future studies should focus on targeted trials incorporating protocolized rhythm assessment, particularly in HFpEF cohorts and patients with implanted devices, to bridge evidence gaps in subclinical arrhythmia identification and clarify direct electrophysiological mechanisms.

## Figures and Tables

**Table 1:** Risk of bias assessment table.

Trial	Randomization Process	Deviations from Intended Interventions	Missing Outcome Data	Outcome Measurement	Selection of Reported Result
SOLOIST-WHF [7]	Low risk	Low risk	Low risk	Low risk	Low risk
EMPEROR- REDUCED [8]	Low risk	Low risk	Low risk	Low risk	Low risk
DAPA-HF [9]	Low risk	Low risk	Low risk	Low risk	Low risk
EMPEROR- PRESERVED [10]	Low risk	Low risk	Low risk	Low risk	Low risk
DELIVER [11]	Low risk	Low risk	Low risk	Low risk	Low risk
DEFINE-HF [12]	Some concerns	Low risk	Low risk	Low risk	Low risk
CHIEF-HF [13]	Low risk	Low risk	Low risk	Low risk	Low risk
PRESERVED-HF [14]	Low risk	Low risk	Low risk	Low risk	Low risk
EMPERIAL- PRESERVED [15]	Low risk	Low risk	Low risk	Low risk	Low risk
EMPERIAL-REDUCED [16]	Low risk	Low risk	Low risk	Low risk	Low risk
EMBRACE-HF [17]	Some concerns	Low risk	Low risk	Low risk	Low risk

**Table 2:** Inclusion and exclusion criteria.

Inclusion Criteria	Exclusion Criteria
(a) Human Studies	(a) Animal Studies
(b) From 2014–2024	(b) >10 years old
(c) English Text	(c) Non-English Texts
(d) Randomized control trials	(d) Case-control, case report, cohort case series, systematic reviews, literature reviews
(e) Patients with heart failure without acute decompensation	(e) Studies involving clinical data other than cardiovascular diseases
	(f) Patients without heart failure/acute decompensated heart failure

**Table 3:** Baseline characteristics: This table presents baseline characteristics from randomized controlled trials comparing SGLT2 inhibitors with placebo in heart failure populations. For each study arm, the table details the number of participants, mean age (years), number of male participants, mean left ventricular ejection fraction (LVEF), and heart failure type (HFrEF: heart failure with reduced ejection fraction, HFpEF: heart failure with preserved ejection fraction). Missing values reflect information not reported in the original articles.

Trial	Group	Number	Age (Mean ± SD)	Males	LVEF	Beta Blocker	Type of Heart Failure	Follow-Up
SOLOIST-WHF [7]	Sotagliflozin	608	68.6 ± 9.5	410	35 ± 14.07	564 (92.8%)	HFrEF and HFpEF	2 years
	Placebo	614	69.3 ± 8.8	400	35 ± 12.6	561 (91.4%)		
EMPEROR-REDUCED [8]	Empagliflozin	1863	67.2 ± 10.8	1426	27.7 ± 6.0	1765 (94.7%)	HFrEF	2.85 years
	Placebo	1867	66.5 ± 11.2	1411	27.2 ± 6.1	1768 (94.7%)		
DAPA-HF [9]	Dapagliflozin	2373	66.2 ± 11	564	31.2 ± 6.7	2278 (96%)	HFrEF	2.317 years
	Placebo	2371	66.5 ± 10.8	545	30.9 ± 6.9	2280 (96.2%)		
EMPEROR-PRESERVED [10]	Empagliflozin	2997	71.8 ± 9.3	1659	54.3 ± 8.8	-	HFpEF	2.858 years
	Placebo	2991	71.9 ± 9.6	1653	54.3 ± 8.8	-		
DELIVER [11]	Dapagliflozin	3131	71.8 ± 9.6	1767	54.0± 8.6	-	HFpEF and mild reduced	3.508 years
	Placebo	3132	71.5 ± 9.5	1749	54.3 ± 8.9	-		
DEFINE-HF [12]	Dapaglifozin	131	62.2 ± 11	95	27.2 ±8.0%	130 (99.2%)	HFrEF	12 weeks
	Placebo	132	60.4 ± 12	98	25.7 ±8.2%	124 (93.9%)		
CHIEF-HF [13]	Canagliflozin	238	62.9 ± 13.15	119	-	-	HFrEF and HFpEF	12 weeks
	Placebo	238	63.8 ± 13.5	132	-	-		
PRESERVED-HF [14]	Dapagliflozin	162	69 ± 5	70	60 ± 5	-	HFpEF	12 weeks
	Placebo	162	71 ± 5	70	60 ± 5	-		
EMPERIAL-PRESERVED [15]	Empagliflozin	157	73 ± 9	87	-	140 (89.2%)	HFpEF	12 weeks
	Placebo	158	73.9 ± 8.6	92	-	141 (89.2%)		
EMPERIAL- REDUCED [16]	Empagliflozin	155	68.7 ± 9.9	121	-	148 (94.9%)	HFrEF	12 weeks
	Placebo	156	69.3 ± 10.6	111	-	147 (94.2%)		
EMBRACE-HF [17]	Empagliflozin	33	69.5 ± 12.0	21	46.7 ± 14.9	29 (87.9%)	HFrEF and HFpEF	12 weeks
	Placebo	32	62.9 ± 13.3	20	40.7± 17.2	29 (90.6%)		

**Table 4:** Summary table: Showing the impact of specific SGLT2i (Dapagliflozin and Empagliflozin) on sudden cardiac death, atrial flutter, bradyarrhythmia and conduction disorders and atrioventricular block.

Drug	Outcome	Relative Risk (RR)	95% CI	*p*-Value	Clinical Implication
Dapagliflozin	Sudden Cardiac Death	0.68	0.47–0.99	0.047	Reduced risk
Dapagliflozin	Atrial Flutter	3.03	1.18–7.75	0.02	Increased risk
Empagliflozin	Bradyarrhythmia and Conduction Disorders	1.68	1.02–2.79	0.04	Increased risk

## Data Availability

All data supporting the findings of this study are directly available in the published references cited within the manuscript. The dataset used for data extraction and analysis is publicly accessible. No new data were generated in this meta-analysis. (https://docs.google.com/spreadsheets/d/10aUI3NaJwwZ68yV70nUlaJOxQ-PFh_Dq1-W4w4dkwB0/edit?usp=sharing) accessed on 25 June 2025.

## Supplementary Materials

The following supporting information can be downloaded at: https://www.mdpi.com/article/10.3390/jcm14155306/s1, Table S1: Search strategies, Figure S1: Risk of ventricular fibrillation, Figure S2: Risk of ventricular tachycardia, Figure S3: Risk of atrial fibrillation, Figure S4: Risk of atrial flutter, Figure S5: Risk of SAN dysfunction, Figure S6: Risk of atrioventricular block, and Figure S7: Risk of intraventricular block.

## Abbreviations

Ventricular arrhythmia (VA), SGLT2 inhibitors (SGLT2i), heart failure with reduced ejection fraction (HFrEF), heart failure with preserved ejection fraction (HFpEF), sudden cardiac death (SCD).
