# Supplementary material for: SGLT2 Inhibitors and the Risk of Arrhythmias in Heart Failure: A Network Meta-Analysis
Source: J Clin Med. 2025 Jul 27;14(15):5306. doi: 10.3390/jcm14155306 (PMC12347573; doi:10.3390/jcm14155306)
Supplement: Supplementary file 1 [file jcm-14-05306-s001.zip › jcm-3765969-supplementary.pdf]

1. Search strategies

Table S1: Search Strategies

Database	Search Strategy	Results
Pubmed	("Sodium-Glucose Transporter 2 Inhibitors"[Mesh] OR "Sodium-Glucose Transporter 2 Inhibitors"[Pharmacological Action] OR "empagliflozin"[Supplementary Concept] OR "dapagliflozin"[Supplementary Concept] OR "canagliflozin"[Supplementary Concept] OR "ertugliflozin"[Supplementary Concept] OR "SGLT2 inhibitors"[All Fields] OR "SGLT-2 inhibitors"[All Fields]) AND ("Heart Failure"[Mesh] OR "heart failure"[All Fields] OR "heart failure with reduced ejection fraction"[All Fields] OR "heart failure with preserved ejection fraction"[All Fields] OR "HFrEF"[All Fields] OR "HFpEF"[All Fields]) AND ("Arrhythmias, Cardiac"[Mesh] OR "cardiac arrhythmia"[All Fields] OR "Tachycardia, Ventricular"[Mesh] OR "ventricular tachycardia"[All Fields] OR "Ventricular Fibrillation"[Mesh] OR "ventricular fibrillation"[All Fields] OR "ventricular arrhythmia"[All Fields] OR "ventricular tachyarrhythmia"[All Fields] OR "torsades de pointes"[All Fields] OR "Death, Sudden, Cardiac"[Mesh] OR "sudden cardiac death"[All Fields] OR "cardiac arrest"[All Fields] OR "Atrial Fibrillation"[Mesh] OR "atrial fibrillation"[All Fields] OR "AFib"[All Fields] OR "Atrial Flutter"[Mesh] OR "atrial flutter"[All Fields] OR "atrial arrhythmia"[All Fields] OR "supraventricular tachycardia"[All Fields] OR "SVT"[All Fields] OR "SVA"[All Fields] OR "PSVT"[All Fields] OR "Heart Block"[Mesh] OR "heart block"[All Fields] OR "AV block"[All Fields] OR "Bundle-Branch Block"[Mesh] OR "bundle branch block"[All Fields] OR "LBBB"[All Fields] OR "RBBB"[All Fields] OR "bradyarrhythmia"[All Fields] OR "bradycardia"[All Fields] OR "trifascicular block"[All Fields])	246 (03/19/2025)<br><br>26<br>Filters applied:<br>Last 10 years<br>Full text<br>RCT<br>Clinical trials
pubmed	("Heart Failure"[Mesh]) AND ("Sodium-Glucose Transporter 2 Inhibitors"[Mesh] OR "Sodium-Glucose Transport Proteins"[Mesh])	1,643 (03/19/2025)<br><br>182<br>Filters applied:<br>Last 10 years<br>Full text<br>RCT<br>Clinical trials

Google scholar	("SGLT2 inhibitor" OR "SGLT-2 inhibitor") AND ("heart failure" OR HFrEF OR HFpEF) AND (arrhythmia OR "atrial fibrillation" OR "atrial flutter" OR "supraventricular tachycardia" OR "SVT" OR "ventricular tachycardia" OR "ventricular fibrillation" OR "sudden cardiac death" OR "cardiac arrest" OR "heart block" OR "bundle branch block" OR "bradyarrhythmia" OR "AV block" OR torsades) AND ("randomized controlled trial" OR "randomized clinical trial" OR RCT or trial)	7,100 (03/19/2025)<br><br>6,760→ 300→ 0(title and abstract screening, removing duplicates)<br>Filters applied:<br>Last 10 years<br>Screened first 15 pages<br><br>Excluding meta analysis, reviews, retrospective studies, not relevant population or outcome
Cochrane	#1 MeSH descriptor: [Sodium-Glucose Transporter 2 Inhibitors] explode all trees<br><br>#2 MeSH descriptor: [Heart Failure] explode all trees<br><br>#3 MeSH descriptor: [Arrhythmias, Cardiac] explode all trees<br><br>#1 and #2 and #3	6 (03/19/2025)<br><br>0<br>Removing duplicates and irrelevant studies
clinicaltrials.gov	Heart failure and SGLT2 inhibitors	196 (03/19/2025)<br><br>27 → 10<br>Filters applied:<br>Completed<br>With results
ScienceDirect	sglt2 inhibitors and heart failure and arrhythmia	1,225 (03/19/2025)<br><br>312 →0 (screening out irrelevant studies to research question)<br>Filters applied:<br>Last 10 years<br>Research articles<br>English

PlosOne	((everything:"sglt2 inhibitor" OR everything:"sglt-2 inhibitor" OR everything:"sodium glucose cotransporter 2" OR everything:"empagliflozin" OR everything:"dapagliflozin" OR everything:"canagliflozin" OR everything:"ertugliflozin")<br>AND<br>(everything:"heart failure" OR everything:"HFrEF" OR everything:"HFpEF")<br>AND<br>((everything:arrhythmia) OR (everything:"ventricular arrhythmia") OR (everything:"atrial fibrillation") OR (everything:"atrial flutter") OR (everything:"ventricular tachycardia") OR (everything:"ventricular fibrillation") OR (everything:"sudden cardiac death") OR (everything:"cardiac arrest") OR (everything:"heart block") OR (everything:"atrioventricular block") OR (everything:"bundle branch block") OR (everything:"bradyarrhythmia") OR (everything:"torsades de pointes")))	78<br>(03/19/2025)<br><br>71 → 0 (after screening title and abstract)<br>Filters applied:<br>Medicine and health sciences<br>Research article

2. Outcomes

Ventricular arrhythmia
Our meta-analysis included five RCTs with 21,916 participants to evaluate ventricular arrhythmia risk with SGLT2 inhibitors. We analyzed all reported cases of ventricular rhythm disturbances including torsades de pointes, ventricular flutter, extrasystole, tachyarrhythmia, fibrillation, tachycardia, and other ventricular arrhythmias. The pooled results showed no significant reduction in ventricular arrhythmia risk with SGLT2 inhibitor treatment versus placebo (RR: 1.02 [0.83–1.25]; I^2 = 0%, p = 0.87).

When stratified by heart failure phenotype, neither patients with preserved ejection fraction (HFpEF, n = 12,238; RR: 0.96 [0.65–1.42]; I^2 = 0%, p = 0.84) nor those with reduced ejection fraction (HFrEF, n = 8,462; RR: 1.05 [0.70–1.58]; I^2 = 64.1%, p = 0.81) showed significant treatment effects, with high heterogeneity was observed in the HFrEF subgroup.

Drug-specific analyses revealed consistently non-significant effects across individual SGLT2 inhibitors. Dapagliflozin (RR: 0.90 [0.66–1.21]; I^2 = 0%, p = 0.48), empagliflozin (RR: 1.15 [0.87–1.54]; I^2 = 25.1%, p = 0.33), and sotagliflozin (RR: 0.87 [0.29–2.56]; p = 0.79).

Network meta-analysis revealed no significant differences in ventricular arrhythmia risk between treatments: dapagliflozin (RR: 0.90 [0.66–1.21]), empagliflozin (RR: 1.15 [0.87–1.54]) and sotagloflozin (RR: 0.87 [0.29–2.56]) . The network showed no heterogeneity or inconsistency (I^2 = 0%, Q = 1.67, p = 0.43). P-score ranking placed dapagliflozin highest (0.71), followed by sotagliflozin (0.61), placebo (0.49), and empagliflozin (0.20). These rankings should be interpreted cautiously given the lack of significant differences.

Figure S1: Risk of Ventricular FIbrillation

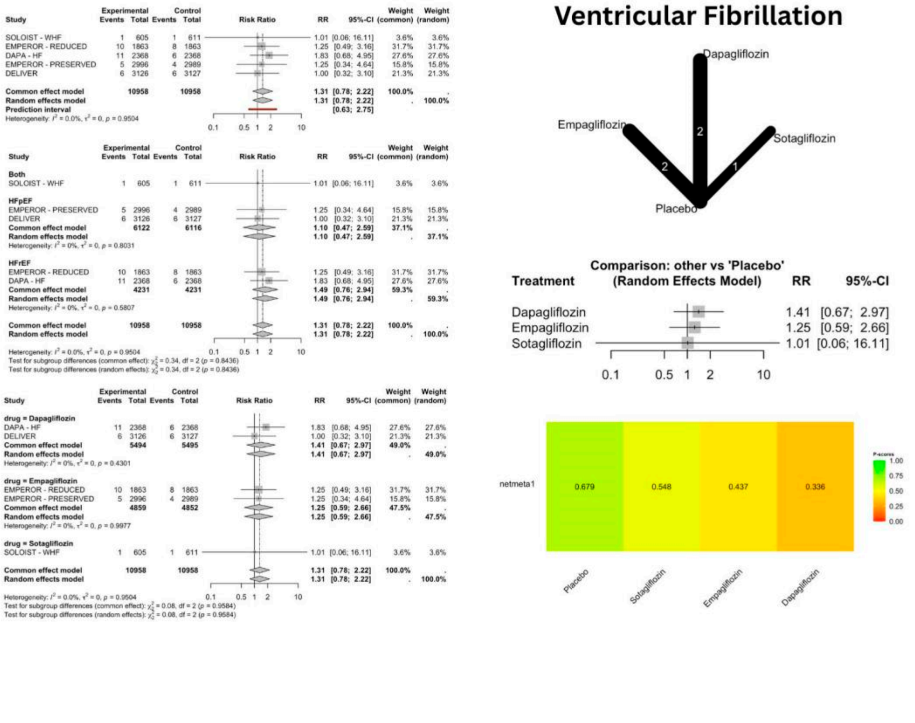

A total of five RCTs (n= 21,916) was analyzed evaluating the risk of ventricular fibrillation. The pooled results demonstrated no significant reduction (RR: 1.31 [0.78–2.22]; I^2 = 0%, p = 0.31). Subgroup analyses on heart failure phenotype, revealed no significant effects of treatment on risk of VF in HFpEF (n = 12,238; RR: 1.1 [0.47–2.59]; I^2 = 0%, p = 0.83) as well as in HFrEF (n = 8,462; RR: 1.49 [0.76–2.94]; I^2 = 0%, p = 0.25). Drug-specific analyses revealed consistently non-significant effects across individual SGLT2 inhibitors: Dapagliflozin (RR: 1.41 [0.67–2.97]; I^2 = 0%, p = 0.37), empagliflozin (RR: 1.25 [0.59–2.66]; I^2 = 0%, p = 0.57), and sotagliflozin (RR: 1.01 [0.06–16.11]; p = 0.99).

Network Meta-Analysis revealed no significant differences vs placebo: Dapagliflozin (RR:1.41 [0.67–2.97], p=0.37), empagliflozin (RR:1.25 [0.59–2.66], p=0.57),and sotagliflozin (RR:1.01 [0.06–16.11], p=0.99), with wide confidence intervals indicating uncertainty. The network showed no heterogeneity (I^2=0%, Q=0.62, p=0.73). P-score rankings (placebo:0.68, sotagliflozin:0.55, empagliflozin:0.44, dapagliflozin:0.34).

Figure S2: Risk of Ventricular Tachycardia

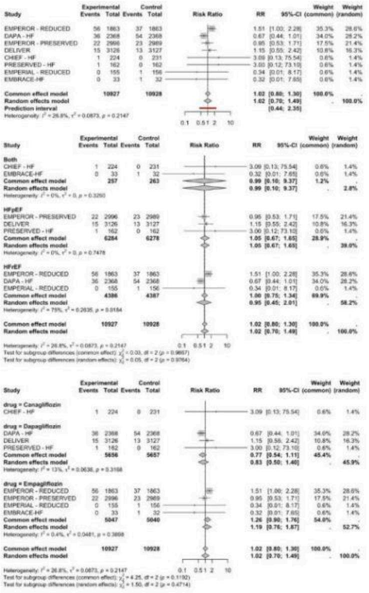

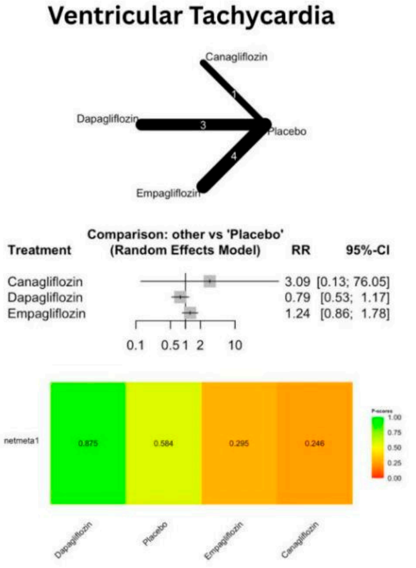

A total of 8 RCTs (n= 21,855) were analyzed evaluating the risk of ventricular tachycardia. The pooled results demonstrated no significant reduction in the risk in fixed effect model (RR: 1.02 [0.80–1.3]; p = 0.90) and random effects model (RR: 1.02 [0.7,1.49], p= 0.93), with a moderate observed heterogeneity (I2= 26.8%). Subgroup analyses on heart failure phenotype, revealed no signifiant effects of SGLT2i on risk of VT in HFpEF (n = 12,562; RR: 1.05 [0.67–1.65]; I^2 = 0%, p = 0.84), as well as in HFrEF (n = 8,773; RR: 0.95 [0.45–2.01]; I^2 = 75%, p = 0.90). Drug-specific analyses revealed consistently non-significant effects across individual SGLT2 inhibitors: Dapagliflozin (RR: 0.77 [0.54–1.11]; I^2 = 13%, p = 0.17), empagliflozin (RR: 1.26 [0.90–1.76]; I^2 = 0.4%, p = 0.17), and canagliflozin (RR: 3.09 [0.13–75.54]; p = 0.49).

Network Meta-Analysis revealed no significant differences versus placebo for dapagliflozin (RR 0.79 [0.53–1.17], p = 0.24), empagliflozin (RR 1.24 [0.86–1.78], p = 0.25), or canagliflozin (RR:3.09 [0.13–76.04], p=0.49). The network exhibited low heterogeneity (I^2 = 5.8%, Q = 5.31, p = 0.38) and consistency between direct/indirect evidence. P-score rankings (dapagliflozin:0.88, placebo:0.58, empagliflozin:0.30, canagliflozin:0.25) reflect non-significant trends.

Atrial Arrhythmia

A total of five RCTs (n= 21,916) was analyzed evaluating the risk of atrial arrhythmia. The analysis incorporated all reported cases of AF, supraventricular tachycardia, paroxysmal supraventricular tachycardia, AFL, atrial tachycardia, and supraventricular arrhythmia. The pooled results demonstrated no significant reduction in the risk (RR: 0.92 [0.67–1.27]; p = 0.63), with high observed heterogeneity(I^2 = 65.3%). Subgroup analyses on heart failure phenotype, in HFpEF (n = 12,238), the risk ratio in fixed effects model was 1.14 (CI: 0.93–1.40; I^2 = 0%, p = 0.19) and random effects model was 1.16 (CI:0.90 – 1.49, p= 0.26) with a moderate heterogeneity (I2= 33.8%), indicating no significant effect. In patients with HFrEF (n = 8,462), the risk ratio was 0.66 (CI: 0.49–0.89; I^2 = 10.4%, p = 0.007), demonstrating significant reduction in risk of atrial arrhythmia. Drug-specific analyses revealed consistently non-significant effects across individual SGLT2 inhibitors: Dapagliflozin (RR: 1.04 [0.61–1.78]; I^2 = 75.6%, p = 0.89), empagliflozin (RR: 0.78 [0.43–1.42]; I^2 = 82.5%, p = 0.42), and sotagliflozin (RR: 1.12 [0.46–2.74]; p = 0.80).

Network Meta-Analysis revealed no significant differences versus placebo for dapagliflozin (RR:1.04 [0.58–1.84], p=0.90), empagliflozin (RR:0.78 [0.44–1.38], p=0.40), and sotagliflozin (RR:1.12 [0.36–3.54], p=0.84). Substantial heterogeneity (I^2=79.7%) indicated variable study results. Tests of heterogeneity within designs confirmed this variability (Q = 9.83, df = 2, p = 0.0073), though no inconsistency was observed between direct and indirect comparisons. P-score rankings (empagliflozin:0.75, placebo:0.44, dapagliflozin:0.42, sotagliflozin:0.39) reflect non-significant trends.

Figure S3: Risk of Atrial Fibrillation

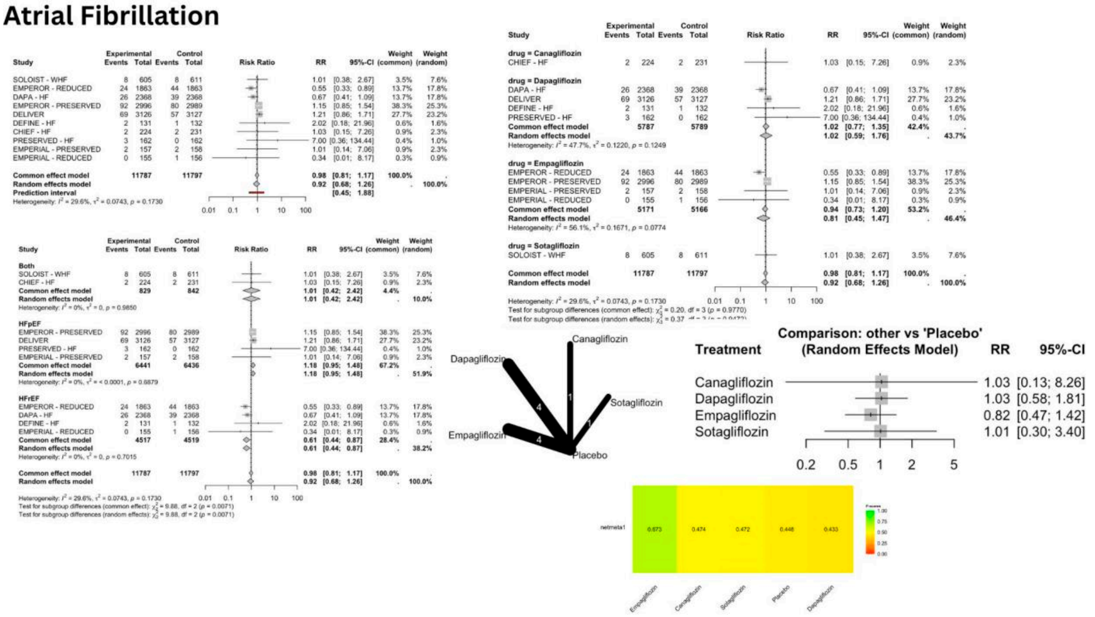

A total of 10 RCTs (n= 23,584) were analyzed evaluating the risk of atrial fibrillation. The pooled results demonstrated no significant reduction in the risk in fixed effects model (RR: 0.98 [0.81–1.17]; p = 0.79) and random effects model (RR: 0.92 [0.68 –1.26], p= 0.62) with a moderate heterogeneity(I^2 = 29.6%). Subgroup analyses on heart failure phenotype in HFpEF (n = 12, 877), the risk ratio showed no significant reduction (RR: 1.18 [0.95–1.48], I2= 0%, p= 0.14) compared to HFrEF (n = 9,036), the risk ratio was 0.61 (CI: 0.44–0.87; I^2 = 0%, p = 0.0053), demonstrating significant reduction. Drug-specific analyses revealed consistently non-significant effects across individual SGLT2 inhibitors: Dapagliflozin (RR: 1.04 [0.61–1.78]; I^2 = 47.7%, p = 0.89 in fixed effects model and RR: 1.02 [0.59 –1.76], p= 0.93 in random effects model), empagliflozin (RR: 0.81 [0.45–1.427]; I^2 = 56.1%, p = 0.49), sotagliflozin (RR: 1.01 [0.38–2.67]; p = 0.98) and canagliflozin (RR: 1.03 [0.15–7.26], p= 0.96).

Network meta-analysis revealed no SGLT2 inhibitors showed significant differences versus placebo: canagliflozin (RR:1.03 [0.13–8.26]), dapagliflozin (RR:1.03 [0.58–1.81]), empagliflozin (RR:0.82 [0.47–1.42]), and sotagliflozin (RR:1.01 [0.30–3.40]). Results were consistent across common/random effects models. Moderate heterogeneity (I^2=52.3%, Q=12.57, p=0.0503) suggested study variability, though no inconsistency existed between direct/indirect evidence. Empagliflozin ranked highest by P-score (0.67), but non-significant differences between SGLT2i.

Figure S4: Risk of Atrial Flutter

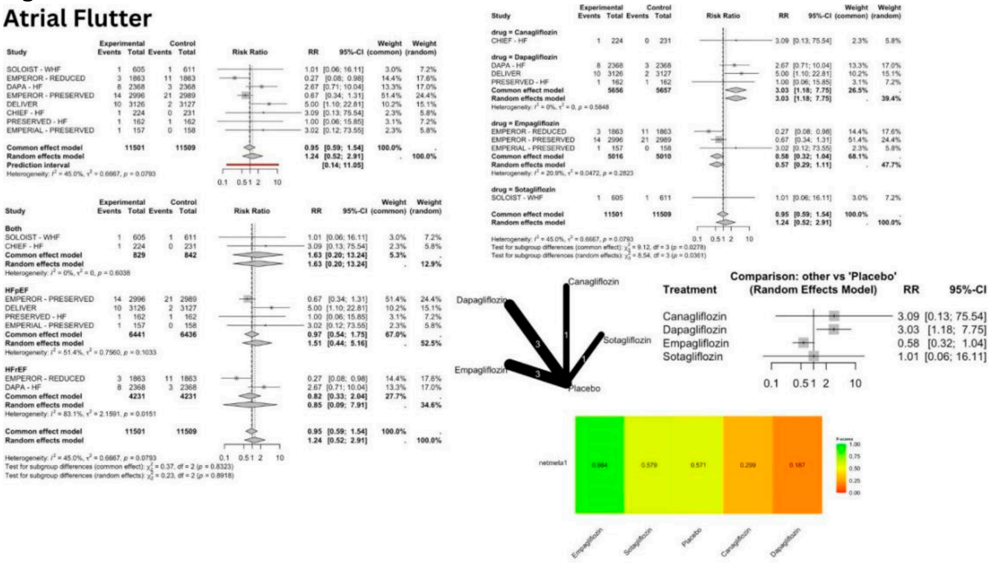

A total of 8 RCTs (n= 23,010) was analyzed evaluating the risk of atrial flutter. The pooled results demonstrated no significant reduction in the risk in fixed effects model (RR: 0.95 [0.59–1.54]; p = 0.84) and random effects model (RR: 1.24 [0.52 –2.91], p= 0.63) with a moderate heterogeneity(I^2 = 45%). Subgroup analyses on heart failure phenotype, revealed no signifiant effects of SGLT2i on risk of AFL in HFpEF (n = 12,877; RR: 1.51 [0.44–5.16], I2= 51.3%, p= 0.52) as well as in HFrEF (n = 8,432; RR: 0.85 [09–7.91]; I^2 = 083.1%, p = 0.88). Drug-specific analyses revealed dapagliflozin had significantly higher risk of AFL by 3.03 (CI: 1.18 - 7.75, I2= 0%, p= 0.02), and the other SGLT2i had no significant effect on risk ratio of AFL: empagliflozin (RR: 0.58 [0.32–1.04], I2= 20.9%, p= 0.07), canagliflozin (RR: 3.09[0.13–75.54], p= 0.49) and sotagliflozin (RR: 1.01[0.06–16.11], p= 0.99)

Network Meta-Analysis showed significantly higher risk on dapagliflozin (RR:3.03 [1.18–7.75], p=0.02), while empagliflozin trended toward reduced risk (RR:0.58 [0.32–1.04], p=0.07). Canagliflozin (RR:3.09 [0.13–75.54], p=0.49) and sotagliflozin (RR:1.01 [0.06–16.11], p=0.99) had inconclusive results due to extreme confidence intervals. Low heterogeneity (I^2=0%, Q=3.6, p=0.46) and consistency between direct/indirect comparisons supported robust findings. P-score rankings (empagliflozin:0.86, sotagliflozin:0.58, placebo:0.57, canagliflozin:0.30, dapagliflozin:0.19) reflect trends but lack clinical significance beyond dapagliflozin's isolated higher risk.

Sudden Cardiac Death

A total of 7 RCTs (n= 22,634) were analyzed evaluating the risk of SCD. The pooled results demonstrated significant reduction in the risk (RR: 0.68 [0.49–0.93]; p = 0.93) with no observed heterogeneity (I^2 = 0%). Subgroup analyses on heart failure phenotype, revealed no signifiant effects of SGLT2i on risk of SCD in HFpEF (n = 12,501, RR: 0.65 [0.41–1.02], I2= 0%, p= 0.06) and in HFrEF (n =

8,462, RR: 0.7 [0.43–1.16]; I^2 = 0%, p = 0.17). Drug-specific analyses revealed dapagliflozin had significantly lower risk of SCD by 0.68 (CI: 0.47 - 0.99), I2= 0%, p= 0.0469), and the other SGLT2i had no significant effect on risk: empagliflozin (RR: 0.64[0.31–1.32], I2= 0%, p= 0.23), canagliflozin (RR: 0.34 [0.01–8.39], p= 0.51) and sotagliflozin (RR: 0.79[0.29–2.10], p= 0.63)

Network meta-analysis revealed dapagliflozin demonstrated a significant 32% risk reduction versus placebo (RR:0.68 [0.47–0.99], p=0.047), while empagliflozin (RR:0.64 [0.31–1.32], p=0.23), canagliflozin (RR:0.34 [0.01–8.39], p=0.51), and sotagliflozin (RR:0.79 [0.29–2.10], p=0.63) showed non-significant effects. Low heterogeneity (I^2=0%, Q=0.8, p=0.85) and consistent direct/indirect comparisons. P-score rankings (canagliflozin:0.68, empagliflozin:0.61, dapagliflozin:0.59) were unreliable for canagliflozin/sotagliflozin due to extreme confidence intervals, emphasizing dapagliflozin’s isolated significance.

Bradyarrhythmia and conduction disorders

A total of five RCTs (n=21,916) were analyzed evaluating the risk of bradyarrhythmia and conduction disorders. The analysis incorporated all reported cases of AV block complete, AV block 2nd degree, Paroxysmal atrioventricular block, bradyarrhythmia, sinus node dysfunction, Sinus arrest, Sinoatrial Block, trifascicular block, Left bundle branch block(LBBB), right bundle branch block (RBBB). The pooled results demonstrated no significant reduction in the risk (RR: 1.22 [0.86–1.73]; p = 0.26), with low heterogeneity(I^2 = 10.4%). Subgroup analyses on heart failure phenotype, revealed no signifiant effects of SGLT2i on risk of bradyarrhythmia and conduction disorders in HFpEF (n = 12,238; RR: 1.14 [0.64–2.04]; I^2 = 53%, p = 0.66) and in HFrEF (n = 8,462; RR: 1.84 [0.81–4.18]; I^2 = 0%, p = 0.15). Drug-specific analyses revealed significantly increased risk in patients on empagliflozin (RR: 1.68 [1.02 - 2.79], I2= 0%, p= 0.04) compared to dapagliflozin (RR: 0.93 [0.56 - 1.55], I2= 0%, p= 0.79) and sotagliflozin (RR: 0.76 [0.17, 3.37], p= 0.72) which are non significant.

On network meta-analysis empagliflozin showed a significantly increased risk versus placebo (RR:1.68 [1.02–2.79], p=0.043), while dapagliflozin (RR:0.93 [0.56–1.55], p=0.79) and sotagliflozin (RR:0.76 [0.17–3.37], p=0.72) demonstrated non-significant effects. Low heterogeneity (I^2=0%, Q=1.45, p=0.48) and consistent direct/indirect comparisons supported robust findings. P-score rankings (sotagliflozin:0.70, dapagliflozin:0.65, placebo:0.58, empagliflozin:0.08) favored sotagliflozin but lack reliability due to wide confidence intervals, emphasizing on empagliflozin’s isolated risk elevation.

Figure S5: Risk of SAN Dysfunction

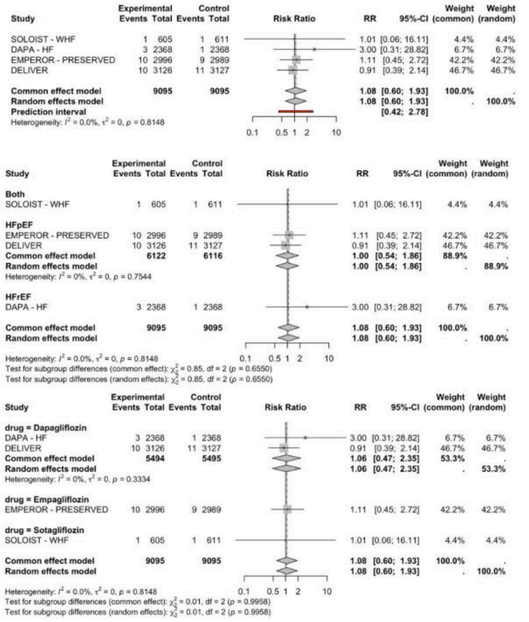

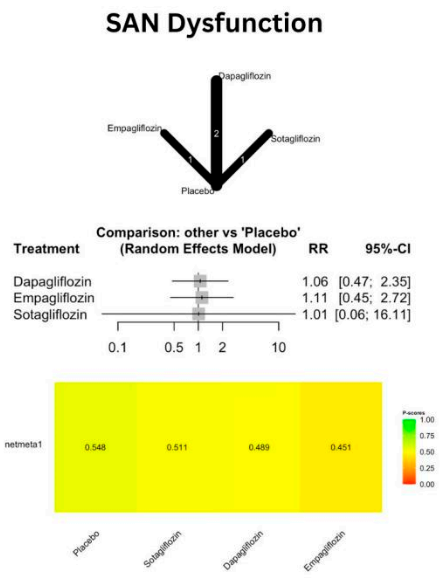

A total of 4 RCTs (n= 18,190) were analyzed evaluating the risk of SAN dysfunction. The pooled results demonstrated no significant reduction in the risk of SAN dysfunction with risk ratio RR: 1.08 (CI: 0.60 – 1.93; I^2 = 0%, p = 0.81). Subgroup analyses on heart failure phenotype, revealed no signifiant effects of SGLT2i on risk of SAN dysfunction in HFpEF (n = 12,238; RR: 1 [0.54–1.86]; I^2 = 0%, p = 0.9976), as well as in HFrEF (n = 4,736; RR: 3 [0.31–28.82]; p = 0.25), consisting of just 1 study. Drug-specific analyses revealed consistently non-significant effects across individual SGLT2 inhibitors: dapagliflozin (RR: 1.06 [0.47–2.35]; I^2 = 0%, p = 0.89), empagliflozin (RR: 1.11 [0.45–2.72];p = 0.82), and sotagliflozin (RR: 1.01 [0.06–16.11]; p = 0.99). These findings indicate that, across individual agents, there was no statistically significant difference in the risk of ventricular fibrillation.

Network Meta-Analysis revealed no significant differences versus placebo: dapagliflozin (RR:1.06 [0.47–2.35], p=0.89), empagliflozin (RR:1.11 [0.45–2.72], p=0.82), and sotagliflozin (RR:1.01 [0.06–16.11], p=0.99). Low heterogeneity (I^2=0%, Q=0.94, p=0.33) and consistent direct/indirect evidence supported robust null findings. P-score rankings (placebo:0.55, sotagliflozin:0.51, dapagliflozin:0.49, empagliflozin:0.45) reflected non-significant trends.

Figure S6: Risk of Atrioventricular Block

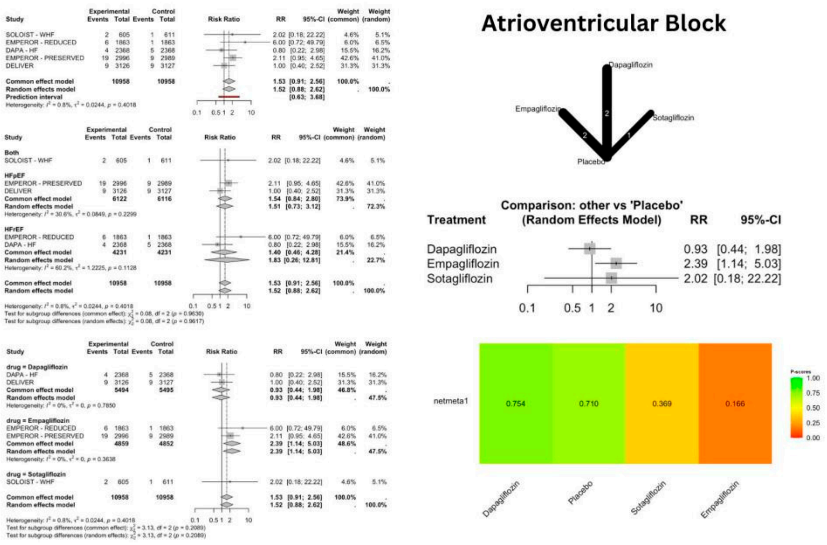

A total of 5 RCTs (n= 21,916) were analyzed evaluating the risk of atrioventricular node block. The analysis incorporated all reported cases of AV block complete, AV block 2nd degree and paroxysmal atrioventricular block. The pooled results demonstrated no significant reduction in the risk (RR: 1.53 [0.91 – 2.56]; I^2 = 0.8%, p = 0.11). Subgroup analyses on heart failure phenotype, revealed no signifiant effects of SGLT2i on risk of AVB in HFpEF (n = 12,238; RR: 1.54 [0.84–2.80]; p = 0.16 in fixed effects model and RR: 1.51 [0.73 - 3.12], p= 0.27 in random effects model with heterogeneity I2= 30.6%) as well as in HFrEF (n = 8,462; RR: 1.83 [0.26–12.81]; p = 0.54 with a high heterogeneity I2= 60.2%). Drug-specific analyses revealed significantly increased risk in patients on empagliflozin (RR: 2.39 [CI: 1.14, 5.03], I2= 0%, p= 0.02) and non significant findings in dapagliflozin (RR: 0.93, [0.44 - 1.98], I2= 0%, p= 0.85) and sotagliflozin (RR: 2.02 [0.18 - 22.22], p= 0.57).

Network meta-analysis revealed significantly increased risk of AVB on empagloflozin (RR: 2.39 [1.14–5.03], p=0.021), while dapagliflozin (RR:0.93 [0.44–1.98], p=0.85) and sotagliflozin (RR:2.02 [0.18–22.22], p=0.57) showed non-significant effects. Minimal heterogeneity (I^2=0%, Q=0.9, p=0.64) and consistent direct/indirect comparisons reinforced robustness. P-score rankings (dapagliflozin:0.75, placebo:0.71, sotagliflozin:0.37, empagliflozin:0.17) favored dapagliflozin.

Figure S7: Risk of Intraventricular Block

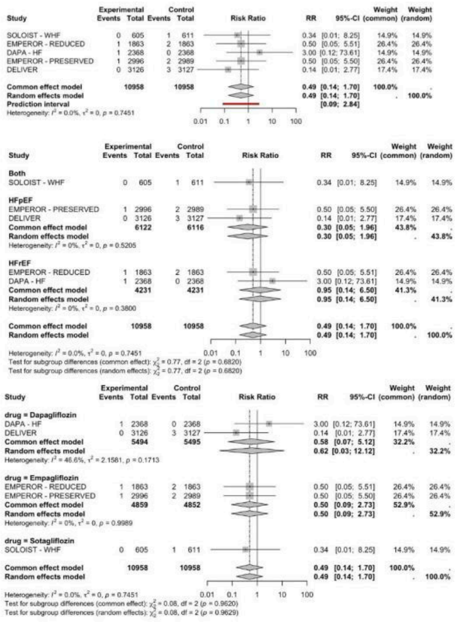

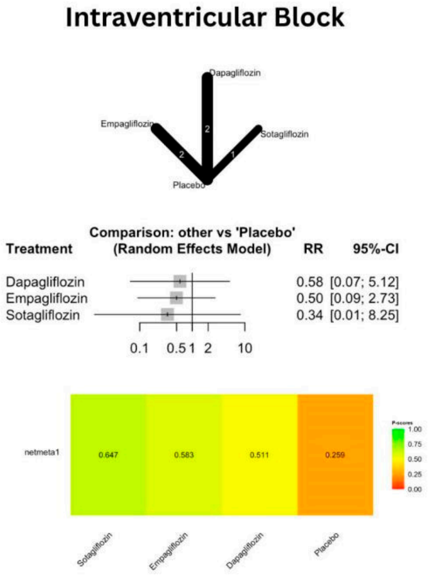

A total of 5 RCTs (n= 21,916) were analyzed evaluating the risk of intraventricular conduction block. The analysis incorporated all reported cases of trifascicular block, Left bundle branch block(LBBB) and right bundle branch block (RBBB). The pooled results demonstrated no significant reduction in the risk (RR: 0.49 [0.14 – 1.7]; I^2 = 0%, p = 0.26). Subgroup analyses on heart failure phenotype, revealed no signifiant effects of SGLT2i in HFpEF (n = 12,238; RR: 0.30 [0.05–1.96]; I^2 = 0%, p = 0.21) as well as in HFrEF (n = 8,462; RR: 0.95 [0.14–6.50]; I2= 0%, p = 0.96). Drug-specific analyses revealed consistently non-significant effects across individual SGLT2 inhibitors: dapagliflozin (RR: 0.58 [0.07 - 5.12], p= 0.63 in fixed effects model and RR: 0.62 [0.03–12.12]; p = 0.75 in random effects model with I2= 46.6%), empagliflozin (RR: 0.5 [0.09–2.73]; I2= 0%, p = 0.42), and sotagliflozin (RR: 0.34 [0.01–8.25]; p = 0.51).

Network meta-analysis revealed no significant differences between SGLT2 inhibitors and placebo: dapagliflozin (RR:0.58 [0.07–5.12], p=0.63), empagliflozin (RR:0.50 [0.09–2.73], p=0.42), and sotagliflozin (RR:0.34 [0.01–8.25], p=0.50). Minimal heterogeneity (I^2=0%, Q=1.87, p=0.39) and consistency between direct/indirect comparisons supported null findings. P-score rankings (sotagliflozin:0.65, empagliflozin:0.58, dapagliflozin:0.51, placebo:0.26) indicated non-significant trends.

3.Inconsistencies

Ventricular Arrhythmia

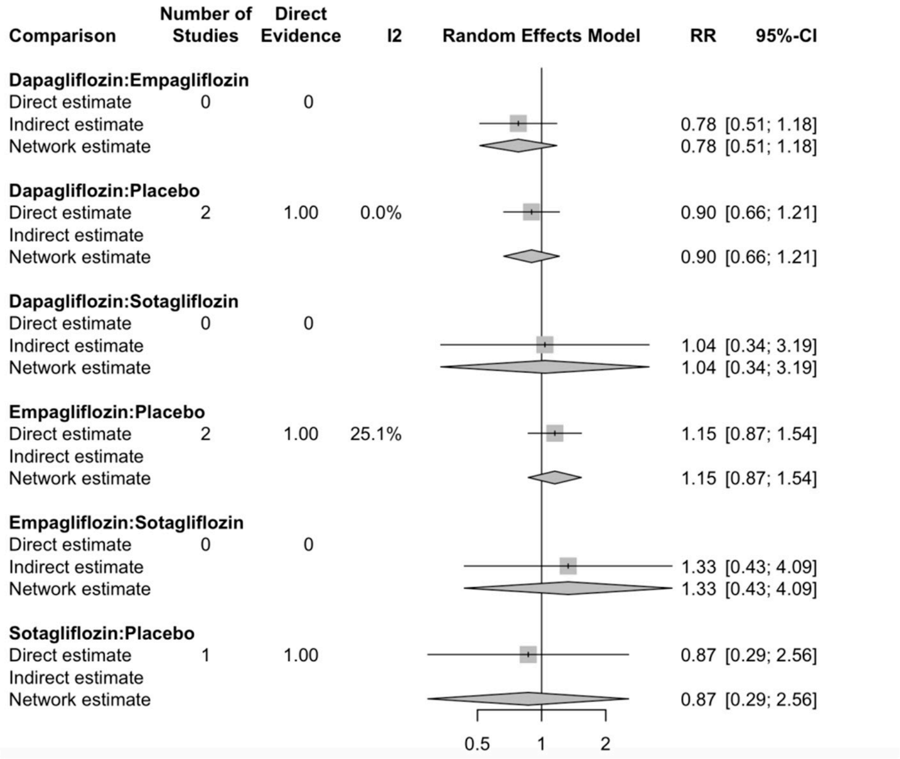

Ventricular Fibrillation

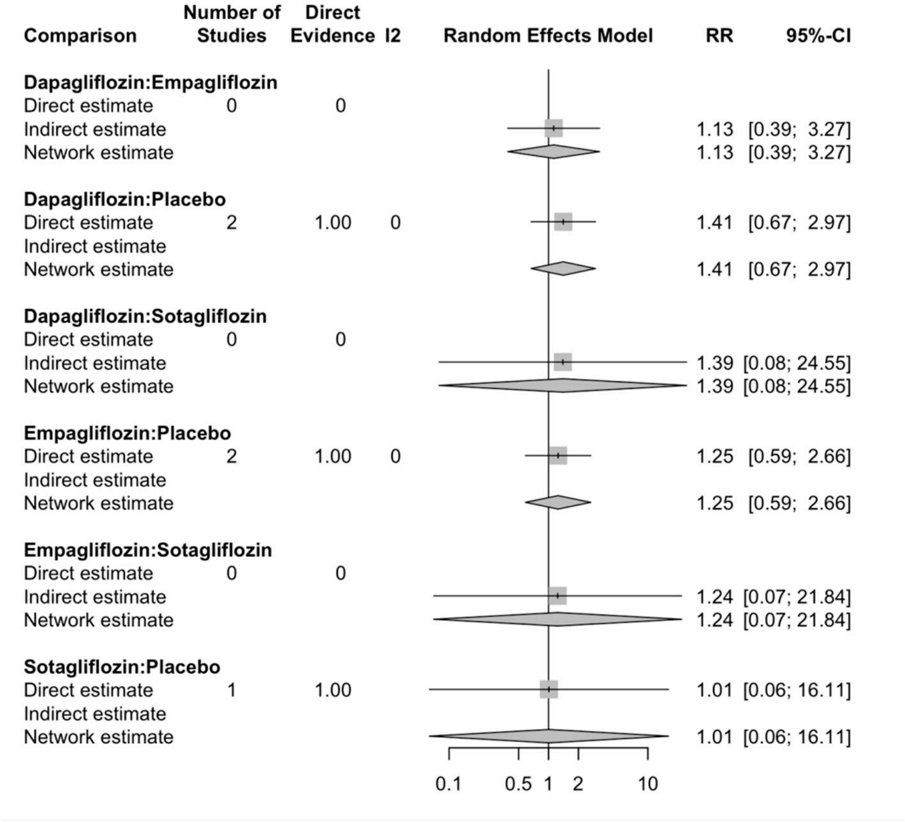

Ventricular Tachycardia

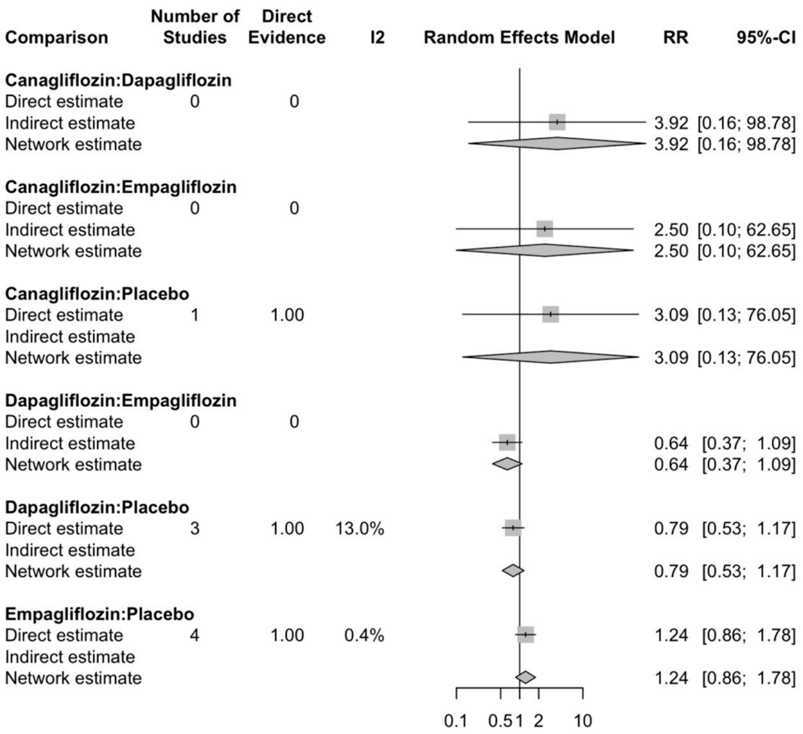

Atrial Arrhythmia

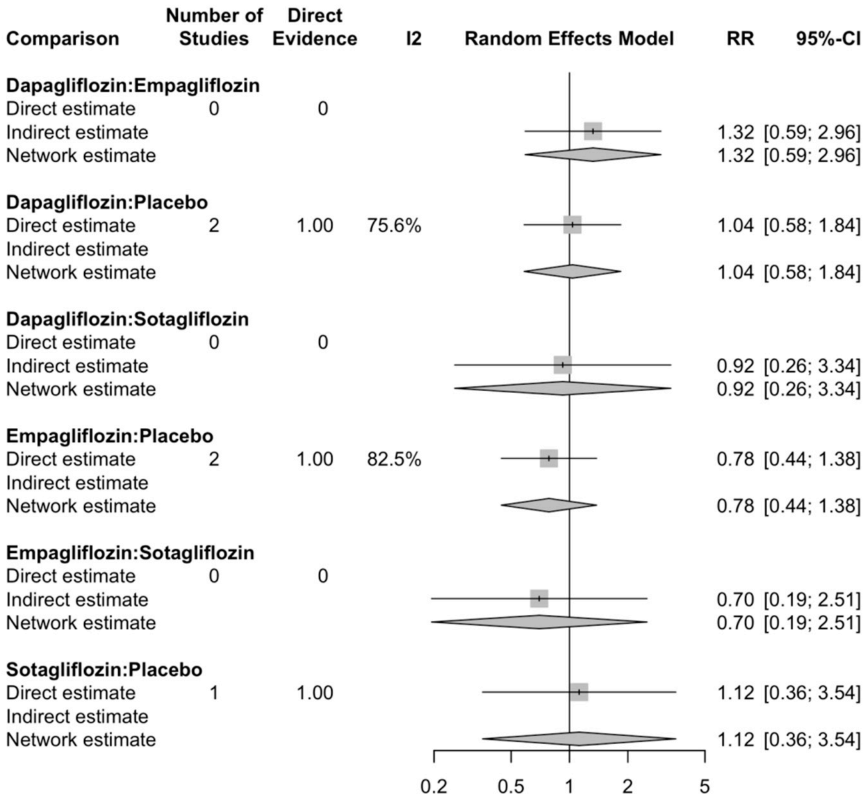

Atrial Fibrillation

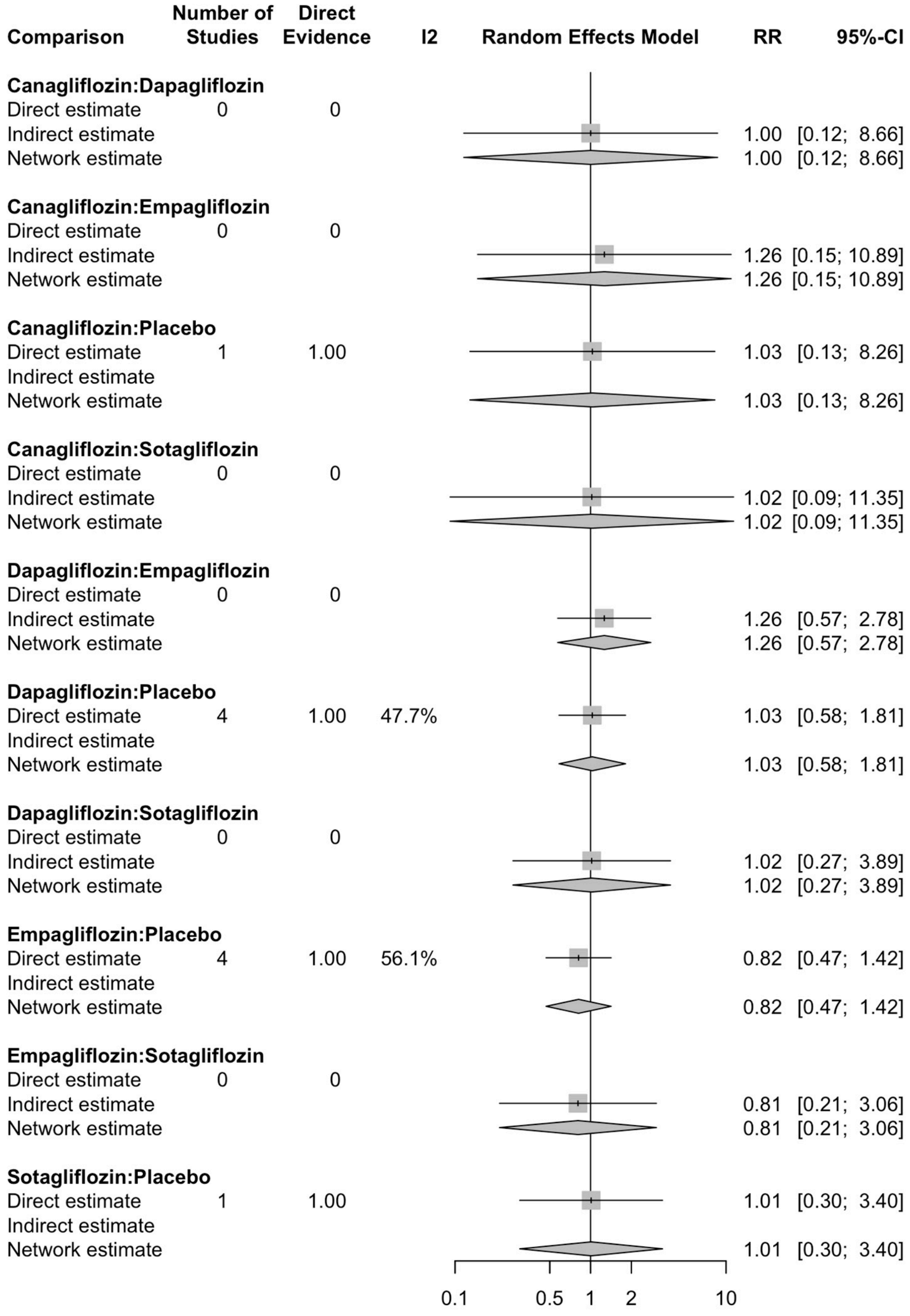

Atrial Flutter

Comparison	Number of Studies	Direct Evidence	I2	RR	95%-CI
**Canagliflozin:Dapagliflozin**					
Direct estimate	0	0			
Indirect estimate				1.02	[0.04; 28.54]
Network estimate				1.02	[0.04; 28.54]
**Canagliflozin:Empagliflozin**					
Direct estimate	0	0			
Indirect estimate				5.34	[0.21; 137.43]
Network estimate				5.34	[0.21; 137.43]
**Canagliflozin:Placebo**					
Direct estimate	1	1.00		3.09	[0.13; 75.54]
Indirect estimate					
Network estimate				3.09	[0.13; 75.54]
**Canagliflozin:Sotagliflozin**					
Direct estimate	0	0			
Indirect estimate				3.06	[0.04; 210.18]
Network estimate				3.06	[0.04; 210.18]
**Dapagliflozin:Empagliflozin**					
Direct estimate	0	0			
Indirect estimate				5.23	[1.73; 15.81]
Network estimate				5.23	[1.73; 15.81]
**Dapagliflozin:Placebo**					
Direct estimate	3	1.00	0.0%	3.03	[1.18; 7.75]
Indirect estimate					
Network estimate				3.03	[1.18; 7.75]
**Dapagliflozin:Sotagliflozin**					
Direct estimate	0	0			
Indirect estimate				3.00	[0.16; 55.86]
Network estimate				3.00	[0.16; 55.86]
**Empagliflozin:Placebo**					
Direct estimate	3	1.00	20.9%	0.58	[0.32; 1.04]
Indirect estimate					
Network estimate				0.58	[0.32; 1.04]
**Empagliflozin:Sotagliflozin**					
Direct estimate	0	0			
Indirect estimate				0.57	[0.03; 9.74]
Network estimate				0.57	[0.03; 9.74]
**Sotagliflozin:Placebo**					
Direct estimate	1	1.00		1.01	[0.06; 16.11]
Indirect estimate					
Network estimate				1.01	[0.06; 16.11]

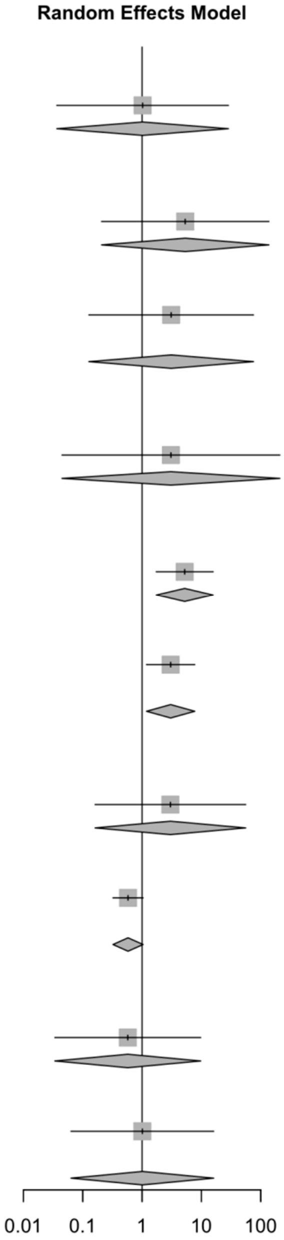

Sudden Cardiac Death

Comparison	Number of Studies	Direct Evidence	I2	RR	95%-CI
**Canagliflozin:Dapagliflozin**					
Direct estimate	0	0			
Indirect estimate				0.50	[0.02; 12.61]
Network estimate				0.50	[0.02; 12.61]
**Canagliflozin:Empagliflozin**					
Direct estimate	0	0			
Indirect estimate				0.54	[0.02; 14.28]
Network estimate				0.54	[0.02; 14.28]
**Canagliflozin:Placebo**					
Direct estimate	1	1.00		0.34	[0.01; 8.39]
Indirect estimate					
Network estimate				0.34	[0.01; 8.39]
**Canagliflozin:Sotagliflozin**					
Direct estimate	0	0			
Indirect estimate				0.44	[0.02; 12.38]
Network estimate				0.44	[0.02; 12.38]
**Dapagliflozin:Empagliflozin**					
Direct estimate	0	0			
Indirect estimate				1.07	[0.47; 2.43]
Network estimate				1.07	[0.47; 2.43]
**Dapagliflozin:Placebo**					
Direct estimate	3	1.00	0	0.68	[0.47; 0.99]
Indirect estimate					
Network estimate				0.68	[0.47; 0.99]
**Dapagliflozin:Sotagliflozin**					
Direct estimate	0	0			
Indirect estimate				0.87	[0.30; 2.48]
Network estimate				0.87	[0.30; 2.48]
**Empagliflozin:Placebo**					
Direct estimate	2	1.00	0	0.64	[0.31; 1.32]
Indirect estimate					
Network estimate				0.64	[0.31; 1.32]
**Empagliflozin:Sotagliflozin**					
Direct estimate	0	0			
Indirect estimate				0.81	[0.24; 2.76]
Network estimate				0.81	[0.24; 2.76]
**Sotagliflozin:Placebo**					
Direct estimate	1	1.00		0.79	[0.29; 2.10]
Indirect estimate					
Network estimate				0.79	[0.29; 2.10]

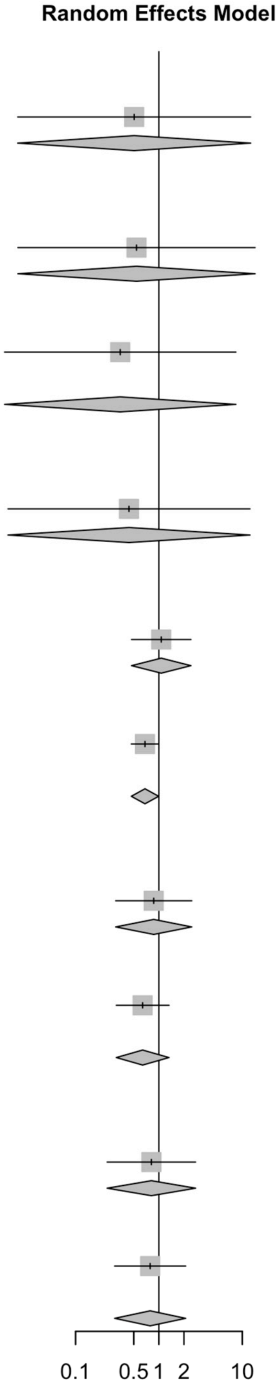

Brady-arrhythmia and conduction disorders

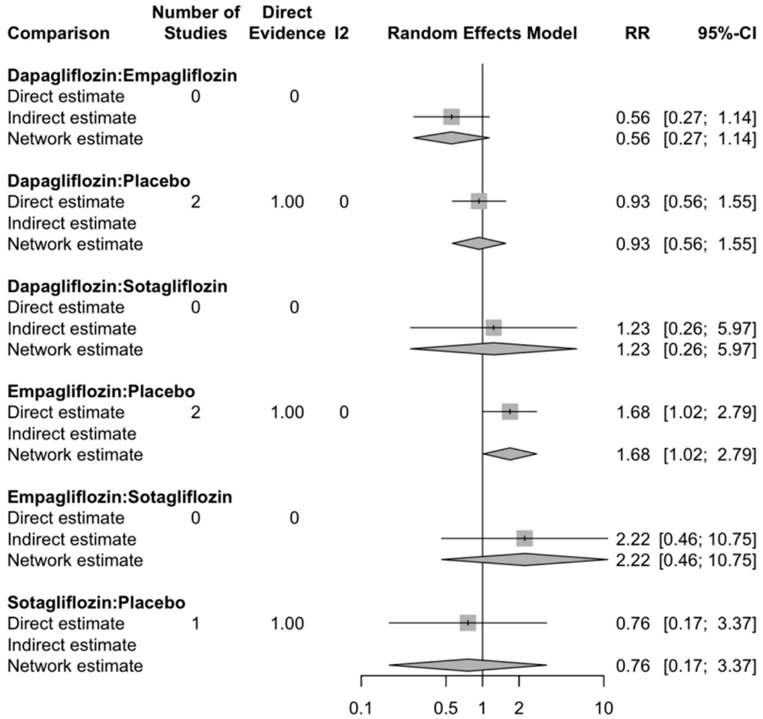

SAN dysfunction

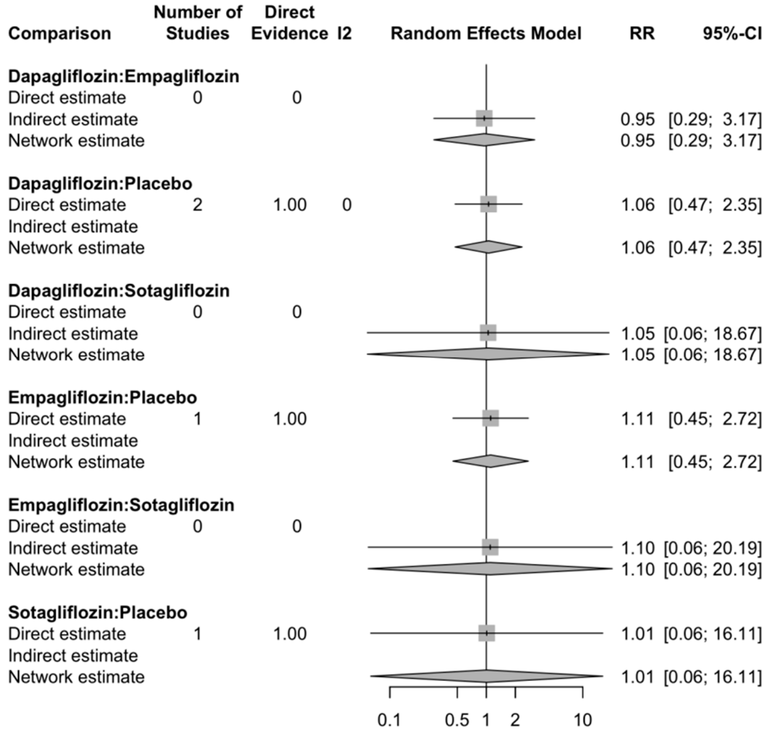

Atrioventricular block

Comparison	Number of Studies	Direct Evidence	I2	RR	95%-CI
**Dapagliflozin:Empagliflozin**					
Direct estimate	0	0			
Indirect estimate				0.39	[0.13; 1.12]
Network estimate				0.39	[0.13; 1.12]
**Dapagliflozin:Placebo**					
Direct estimate	2	1.00	0	0.93	[0.44; 1.98]
Indirect estimate					
Network estimate				0.93	[0.44; 1.98]
**Dapagliflozin:Sotagliflozin**					
Direct estimate	0	0			
Indirect estimate				0.46	[0.04; 5.68]
Network estimate				0.46	[0.04; 5.68]
**Empagliflozin:Placebo**					
Direct estimate	2	1.00	0	2.39	[1.14; 5.03]
Indirect estimate					
Network estimate				2.39	[1.14; 5.03]
**Empagliflozin:Sotagliflozin**					
Direct estimate	0	0			
Indirect estimate				1.19	[0.10; 14.59]
Network estimate				1.19	[0.10; 14.59]
**Sotagliflozin:Placebo**					
Direct estimate	1	1.00		2.02	[0.18; 22.22]
Indirect estimate					
Network estimate				2.02	[0.18; 22.22]

Random Effects Model

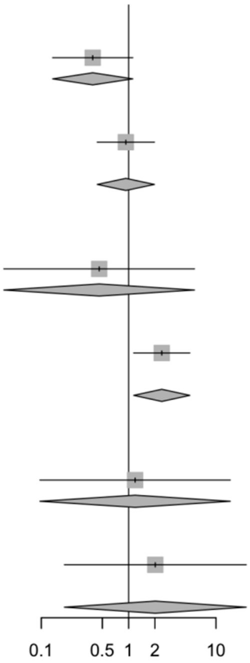

Intraventricular conduction disorder

Comparison	Number of Studies	Direct Evidence	I2	RR	95%-CI
**Dapagliflozin:Empagliflozin**					
Direct estimate	0	0			
Indirect estimate				1.17	[0.07; 18.39]
Network estimate				1.17	[0.07; 18.39]
**Dapagliflozin:Placebo**					
Direct estimate	2	1.00	46.6%	0.58	[0.07; 5.12]
Indirect estimate					
Network estimate				0.58	[0.07; 5.12]
**Dapagliflozin:Sotagliflozin**					
Direct estimate	0	0			
Indirect estimate				1.73	[0.04; 82.74]
Network estimate				1.73	[0.04; 82.74]
**Empagliflozin:Placebo**					
Direct estimate	2	1.00	0.0%	0.50	[0.09; 2.73]
Indirect estimate					
Network estimate				0.50	[0.09; 2.73]
**Empagliflozin:Sotagliflozin**					
Direct estimate	0	0			
Indirect estimate				1.48	[0.04; 55.44]
Network estimate				1.48	[0.04; 55.44]
**Sotagliflozin:Placebo**					
Direct estimate	1	1.00		0.34	[0.01; 8.25]
Indirect estimate					
Network estimate				0.34	[0.01; 8.25]

Random Effects Model

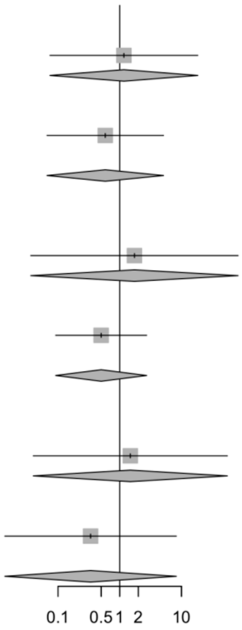

4.League Tables

Ventricular Arrhythmia

League table (common effects model):

```
      Dapagliflozin                    . 0.8972 [0.6641; 1.2120]
 0.7776 [0.5128; 1.1791]         Empagliflozin 1.1537 [0.8652; 1.5385]
 0.8972 [0.6641; 1.2120] 1.1537 [0.8652; 1.5385]             Placebo
 1.0364 [0.3363; 3.1941] 1.3328 [0.4339; 4.0937] 1.1552 [0.3905; 3.4175]


            .
            .
 1.1552 [0.3905; 3.4175]
      Sotagliflozin
```

League table (random effects model):

```
      Dapagliflozin                    . 0.8972 [0.6641; 1.2120]
 0.7776 [0.5128; 1.1791]         Empagliflozin 1.1537 [0.8652; 1.5385]
 0.8972 [0.6641; 1.2120] 1.1537 [0.8652; 1.5385]             Placebo
 1.0364 [0.3363; 3.1941] 1.3328 [0.4339; 4.0937] 1.1552 [0.3905; 3.4175]


            .
            .
 1.1552 [0.3905; 3.4175]
      Sotagliflozin
```

Ventricular Fibrillation

League table (common effects model):

```
       Dapagliflozin                    . 1.4081 [0.6678;  2.9694]
 1.1274 [0.3893;  3.2650]          Empagliflozin 1.2490 [0.5855;  2.6646]
 1.4081 [0.6678;  2.9694] 1.2490 [0.5855;  2.6646]              Placebo
 1.3943 [0.0792; 24.5488] 1.2368 [0.0700; 21.8412] 0.9902 [0.0621; 15.7945]


            .
            .
 0.9902 [0.0621; 15.7945]
       Sotagliflozin
```

League table (random effects model):

```
       Dapagliflozin                    . 1.4081 [0.6678;  2.9694]
 1.1274 [0.3893;  3.2650]          Empagliflozin 1.2490 [0.5855;  2.6646]
 1.4081 [0.6678;  2.9694] 1.2490 [0.5855;  2.6646]              Placebo
 1.3943 [0.0792; 24.5488] 1.2368 [0.0700; 21.8412] 0.9902 [0.0621; 15.7945]


            .
            .
 0.9902 [0.0621; 15.7945]
       Sotagliflozin
```

Ventricular Tachycardia

League table (common effects model):

```
         Canagliflozin                    .                    .
 3.9939 [0.1603; 99.5349]          Dapagliflozin                   .
 2.4541 [0.0988; 60.9633] 0.6145 [0.3762;  1.0037]          Empagliflozin
 3.0935 [0.1267; 75.5394] 0.7746 [0.5395;  1.1120] 1.2606 [0.9047;  1.7564]

 3.0935 [0.1267; 75.5394]
 0.7746 [0.5395;  1.1120]
 1.2606 [0.9047;  1.7564]
              Placebo
```

League table (random effects model):

```
         Canagliflozin                    .                    .
 3.9212 [0.1557; 98.7814]          Dapagliflozin                   .
 2.4962 [0.0995; 62.6491] 0.6366 [0.3717;  1.0903]          Empagliflozin
 3.0935 [0.1258; 76.0527] 0.7889 [0.5309;  1.1723] 1.2393 [0.8610;  1.7838]

 3.0935 [0.1258; 76.0527]
 0.7889 [0.5309;  1.1723]
 1.2393 [0.8610;  1.7838]
              Placebo
```

Atrial Arrhythmia

League table (common effects model):

```
        Dapagliflozin                    . 1.0918 [0.8432; 1.4136]
 1.2435 [0.8846; 1.7480]          Empagliflozin 0.8780 [0.7033; 1.0961]
 1.0918 [0.8432; 1.4136] 0.8780 [0.7033; 1.0961]               Placebo
 0.9729 [0.3838; 2.4662] 0.7824 [0.3116; 1.9645] 0.8912 [0.3647; 2.1777]


                   .
                   .
 0.8912 [0.3647; 2.1777]
        Sotagliflozin
```

League table (random effects model):

```
        Dapagliflozin                    . 1.0359 [0.5830; 1.8407]
 1.3224 [0.5901; 2.9635]          Empagliflozin 0.7833 [0.4447; 1.3798]
 1.0359 [0.5830; 1.8407] 0.7833 [0.4447; 1.3798]               Placebo
 0.9231 [0.2555; 3.3351] 0.6981 [0.1940; 2.5122] 0.8912 [0.2826; 2.8106]


                   .
                   .
 0.8912 [0.2826; 2.8106]
        Sotagliflozin
```

Atrial Fibrillation

League table (common effects model):

Canagliflozin . .
1.0100 [0.1407; 7.2529] Dapagliflozin .
1.0995 [0.1537; 7.8632] 1.0886 [0.7473; 1.5857] Empagliflozin
1.0313 [0.1465; 7.2580] 1.0210 [0.7712; 1.3518] 0.9379 [0.7301; 1.2049]
1.0211 [0.1153; 9.0394] 1.0110 [0.3671; 2.7845] 0.9287 [0.3399; 2.5377]

1.0312 [0.1465; 7.2580] .
1.0210 [0.7712; 1.3518] .
0.9379 [0.7301; 1.2049] .
Placebo 0.9902 [0.3740; 2.6212]
0.9902 [0.3740; 2.6212] Sotagliflozin

League table (random effects model):

Canagliflozin . .
1.0018 [0.1159; 8.6556] Dapagliflozin .
1.2644 [0.1468; 10.8899] 1.2622 [0.5723; 2.7836] Empagliflozin
1.0312 [0.1287; 8.2629] 1.0294 [0.5849; 1.8120] 0.8156 [0.4691; 1.4180]
1.0211 [0.0918; 11.3530] 1.0193 [0.2674; 3.8852] 0.8076 [0.2130; 3.0623]

1.0312 [0.1287; 8.2629] .
1.0294 [0.5849; 1.8120] .
0.8156 [0.4691; 1.4180] .
Placebo 0.9902 [0.2945; 3.3295]
0.9902 [0.2945; 3.3295] Sotagliflozin

Atrial Flutter

League table (common effects model):

Canagliflozin . .
1.0211 [0.0365; 28.5412] Dapagliflozin .
5.3360 [0.2072; 137.4286] 5.2256 [1.7277; 15.8053] Empagliflozin
3.0935 [0.1267; 75.5394] 3.0295 [1.1847; 7.7472] 0.5797 [0.3227; 1.0417]
3.0632 [0.0446; 210.1829] 2.9998 [0.1611; 55.8623] 0.5741 [0.0338; 9.7358]

3.0935 [0.1267; 75.5394] .
3.0295 [1.1847; 7.7472] .
0.5797 [0.3227; 1.0417] .
Placebo 0.9902 [0.0621; 15.7945]
0.9902 [0.0621; 15.7945] Sotagliflozin

League table (random effects model):

Canagliflozin . .
1.0211 [0.0365; 28.5412] Dapagliflozin .
5.3360 [0.2072; 137.4286] 5.2256 [1.7277; 15.8053] Empagliflozin
3.0935 [0.1267; 75.5394] 3.0295 [1.1847; 7.7472] 0.5797 [0.3227; 1.0417]
3.0632 [0.0446; 210.1829] 2.9998 [0.1611; 55.8623] 0.5741 [0.0338; 9.7358]

3.0935 [0.1267; 75.5394] .
3.0295 [1.1847; 7.7472] .
0.5797 [0.3227; 1.0417] .
Placebo 0.9902 [0.0621; 15.7945]
0.9902 [0.0621; 15.7945] Sotagliflozin

Sudden Cardiac Death
League table (common effects model):

Canagliflozin . .
0.5049 [0.0202; 12.6092] Dapagliflozin .
0.5387 [0.0203; 14.2774] 1.0669 [0.4693; 2.4254] Empagliflozin
0.3437 [0.0141; 8.3933] 0.6808 [0.4659; 0.9948] 0.6381 [0.3080; 1.3220]
0.4376 [0.0155; 12.3810] 0.8667 [0.3026; 2.4817] 0.8123 [0.2393; 2.7574]

0.3437 [0.0141; 8.3933] .
0.6808 [0.4659; 0.9948] .
0.6381 [0.3080; 1.3220] .
Placebo 1.2731 [0.4772; 3.3965]
1.2731 [0.4772; 3.3965] Sotagliflozin

League table (random effects model):

Canagliflozin . .
0.5049 [0.0202; 12.6092] Dapagliflozin .
0.5387 [0.0203; 14.2774] 1.0669 [0.4693; 2.4254] Empagliflozin
0.3437 [0.0141; 8.3933] 0.6808 [0.4659; 0.9948] 0.6381 [0.3080; 1.3220]
0.4376 [0.0155; 12.3810] 0.8667 [0.3026; 2.4817] 0.8123 [0.2393; 2.7574]

0.3437 [0.0141; 8.3933] .
0.6808 [0.4659; 0.9948] .
0.6381 [0.3080; 1.3220] .
Placebo 1.2731 [0.4772; 3.3965]
1.2731 [0.4772; 3.3965] Sotagliflozin

Bradyarrhythmia and conduction disorders
League table (common effects model):

Dapagliflozin . 0.9345 [0.5628; 1.5518]
0.5551 [0.2713; 1.1359] Empagliflozin 1.6834 [1.0156; 2.7902]
0.9345 [0.5628; 1.5518] 1.6834 [1.0156; 2.7902] Placebo
1.2338 [0.2550; 5.9691] 2.2224 [0.4596; 10.7461] 1.3202 [0.2967; 5.8740]

.
.
1.3202 [0.2967; 5.8740]
Sotagliflozin

League table (random effects model):

Dapagliflozin . 0.9345 [0.5628; 1.5518]
0.5551 [0.2713; 1.1359] Empagliflozin 1.6834 [1.0156; 2.7902]
0.9345 [0.5628; 1.5518] 1.6834 [1.0156; 2.7902] Placebo
1.2338 [0.2550; 5.9691] 2.2224 [0.4596; 10.7461] 1.3202 [0.2967; 5.8740]

.

.
1.3202 [0.2967; 5.8740]
Sotagliflozin

SAN dysfunction
League table (common effects model):

Dapagliflozin . 1.0556 [0.4745; 2.3488]
0.9523 [0.2859; 3.1723] Empagliflozin 1.1085 [0.4511; 2.7241]
1.0556 [0.4745; 2.3488] 1.1085 [0.4511; 2.7241] Placebo
1.0453 [0.0585; 18.6709] 1.0976 [0.0597; 20.1856] 0.9902 [0.0621; 15.7945]

.
.
0.9902 [0.0621; 15.7945]
Sotagliflozin

League table (random effects model):

Dapagliflozin . 1.0556 [0.4745; 2.3488]
0.9523 [0.2859; 3.1723] Empagliflozin 1.1085 [0.4511; 2.7241]
1.0556 [0.4745; 2.3488] 1.1085 [0.4511; 2.7241] Placebo
1.0453 [0.0585; 18.6709] 1.0976 [0.0597; 20.1856] 0.9902 [0.0621; 15.7945]

.
.
0.9902 [0.0621; 15.7945]
Sotagliflozin

Atrioventricular block
League table (common effects model):

Dapagliflozin . 0.9291 [0.4367; 1.9768]
0.3880 [0.1347; 1.1176] Empagliflozin 2.3950 [1.1412; 5.0263]
0.9291 [0.4367; 1.9768] 2.3950 [1.1412; 5.0263] Placebo
0.4600 [0.0372; 5.6823] 1.1857 [0.0964; 14.5873] 0.4951 [0.0450; 5.4456]

.
.
0.4951 [0.0450; 5.4456]
Sotagliflozin

League table (random effects model):

Dapagliflozin . 0.9291 [0.4367; 1.9768]
0.3880 [0.1347; 1.1176] Empagliflozin 2.3950 [1.1412; 5.0263]
0.9291 [0.4367; 1.9768] 2.3950 [1.1412; 5.0263] Placebo
0.4600 [0.0372; 5.6823] 1.1857 [0.0964; 14.5873] 0.4951 [0.0450; 5.4456]

.
.
0.4951 [0.0450; 5.4456]
Sotagliflozin

Intraventriuclar conduction disorder

League table (common effects model):

Dapagliflozin . 0.5824 [0.0662; 5.1219]
1.1662 [0.0740; 18.3873] Empagliflozin 0.4994 [0.0915; 2.7253]
0.5824 [0.0662; 5.1219] 0.4994 [0.0915; 2.7253] Placebo
1.7301 [0.0362; 82.7429] 1.4835 [0.0397; 55.4401] 2.9706 [0.1213; 72.7760]

.
.
2.9706 [0.1213; 72.7760]
Sotagliflozin

League table (random effects model):

Dapagliflozin . 0.5824 [0.0662; 5.1219]
1.1662 [0.0740; 18.3873] Empagliflozin 0.4994 [0.0915; 2.7253]
0.5824 [0.0662; 5.1219] 0.4994 [0.0915; 2.7253] Placebo
1.7301 [0.0362; 82.7429] 1.4835 [0.0397; 55.4401] 2.9706 [0.1213; 72.7760]

.
.
2.9706 [0.1213; 72.7760]
Sotagliflozin
